# CCL24 regulates biliary inflammation and fibrosis in primary sclerosing cholangitis

**DOI:** 10.1172/jci.insight.162270

**Published:** 2023-06-22

**Authors:** Raanan Greenman, Michal Segal-Salto, Neta Barashi, Ophir Hay, Avi Katav, Omer Levi, Ilan Vaknin, Revital Aricha, Sarit Aharoni, Tom Snir, Inbal Mishalian, Devorah Olam, Johnny Amer, Ahmad Salhab, Rifaat Safadi, Yaakov Maor, Palak Trivedi, Christopher J. Weston, Francesca Saffioti, Andrew Hall, Massimo Pinzani, Douglas Thorburn, Amnon Peled, Adi Mor

**Affiliations:** 1 Chemomab Therapeutics Ltd., Tel Aviv, Israel.; 2 Gene Therapy Institute, Hadassah Hebrew University Medical Center, Faculty of Medicine, Hebrew University of Jerusalem, Jerusalem, Israel.; 3 Institute of Gastroenterology and Liver Diseases, Department of Medicine, Hadassah Hebrew University Hospital, Jerusalem, Israel.; 4 Institute of Gastroenterology and Hepatology, Kaplan Medical Center, Rehovot, Israel.; 5 National Institute for Health and Care Research Biomedical Research Unit and Centre for Liver Research, University of Birmingham, Birmingham, United Kingdom.; 6 University College London Institute for Liver and Digestive Health, London, United Kingdom.; 7 Sheila Sherlock Liver Centre, Royal Free London NHS Foundation Trust, London, United Kingdom.; 8 Oxford University Hospitals NHS Foundation Trust, Oxford, United Kingdom.

**Keywords:** Hepatology, Inflammation, Chemokines, Fibrosis, Macrophages

## Abstract

ˆCCL24 is a pro-fibrotic, pro-inflammatory chemokine expressed in several chronic fibrotic diseases. In the liver, CCL24 plays a role in fibrosis and inflammation, and blocking CCL24 led to reduced liver injury in experimental models. We studied the role of CCL24 in primary sclerosing cholangitis (PSC) and evaluated the potential therapeutic effect of blocking CCL24 in this disease. Multidrug resistance gene 2–knockout (Mdr2^–/–^) mice demonstrated CCL24 expression in liver macrophages and were used as a relevant experimental PSC model. CCL24-neutralizing monoclonal antibody, CM-101, significantly improved inflammation, fibrosis, and cholestasis-related markers in the biliary area. Moreover, using spatial transcriptomics, we observed reduced proliferation and senescence of cholangiocytes following CCL24 neutralization. Next, we demonstrated that CCL24 expression was elevated under pro-fibrotic conditions in primary human cholangiocytes and macrophages, and it induced proliferation of primary human hepatic stellate cells and cholangiocytes, which was attenuated following CCL24 inhibition. Correspondingly, CCL24 was found to be highly expressed in liver biopsies of patients with PSC. CCL24 serum levels correlated with Enhanced Liver Fibrosis score, most notably in patients with high alkaline phosphatase levels. These results suggest that blocking CCL24 may have a therapeutic effect in patients with PSC by reducing liver inflammation, fibrosis, and cholestasis.

## Graphical abstract

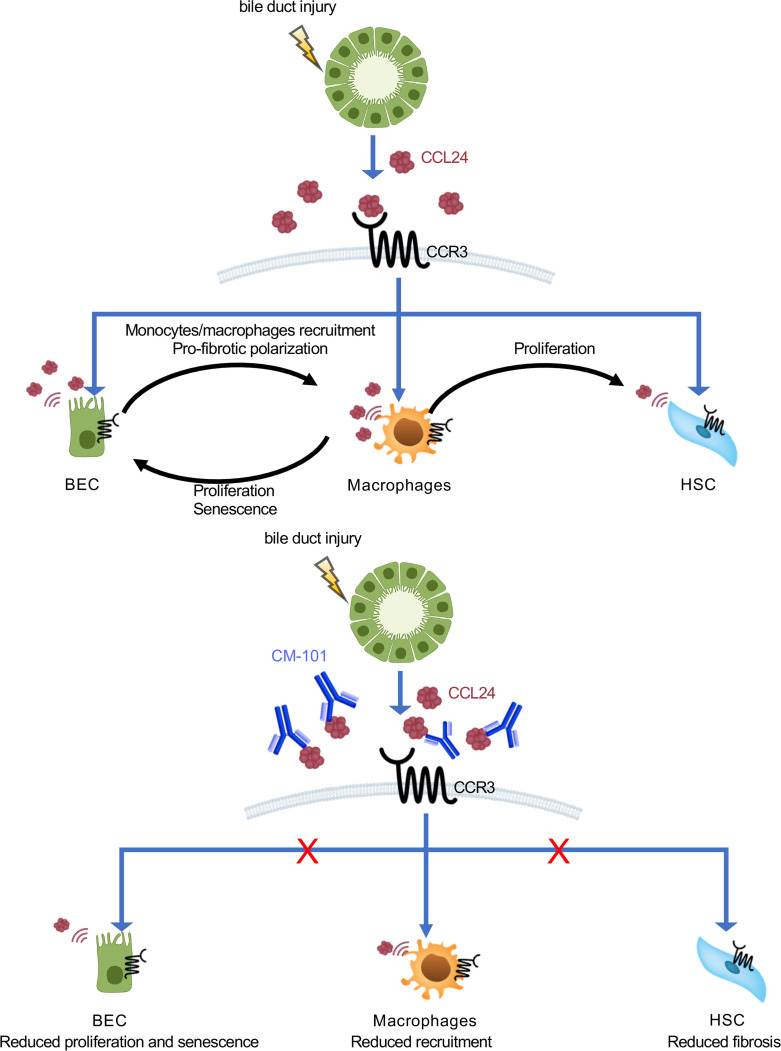

## Introduction

Primary sclerosing cholangitis (PSC) is a chronic, idiopathic, cholestatic liver disease characterized by peribiliary inflammation and fibrosis. It is a progressive, debilitating disorder that can lead to decompensated cirrhosis, hepatobiliary malignancies, and a need for lifesaving liver transplantation. Liver fibrosis and development of cirrhosis are the results of liver damage induced by reactive cholangiocytes accompanied by inflammatory cell infiltration and extensive deposition of extracellular matrix (ECM) proteins by myofibroblasts (1, 2). The etiology and pathogenesis of PSC involve many unresolved questions and remain scientific and clinical challenges (3–5).

Chemokines are a group of small signaling proteins, thought to be involved in the pathogenesis of multiple inflammatory and fibrotic diseases. They play a key role in orchestrating the influx of immune cells into diseased organs (6–10). In the liver, chemokines are involved in the development of inflammation and a chronic/aberrant “wound healing” response, and they can influence the balance between an acute immune response and a chronic unresolved inflammatory process that supports the development of fibrosis (11–13). In addition to their effect on immune regulation, chemokine receptors were also found to be expressed by liver myofibroblasts, indicating a possible direct effect of chemokines on liver myofibroblast activation (14, 15).

CCL24 is a member of the CC chemokine ligand family that signals through CCR3, a G protein–coupled receptor found on the surface of various target cells (16). Unlike other chemokines that recruit immune cells through activation or deactivation of several chemokine receptors, CCL24 solely binds to its receptor, CCR3, and uniquely promotes activities that affect fibrotic processes. Due to the high expression of CCR3 on eosinophils, CCL24 has been mainly studied in diseases that have a known eosinophilic component (17–20). More recently, it has been demonstrated that CCL24 plays an important role in chronic inflammation and fibrosis in several organs, including liver, skin, and lung. Kohan et al. showed that CCL24 can stimulate human lung fibroblast proliferation and collagen synthesis, contributing to the development of lung fibrosis (21). In several independent studies, CCL24 was shown to be upregulated and associated with disease severity in patients with idiopathic pulmonary fibrosis and systemic sclerosis (22). Moreover, blocking CCL24 with an anti-CCL24 monoclonal antibody in a systemic sclerosis preclinical model resulted in reduction of both skin and lung inflammation and fibrosis (23).

CCL24 is specifically involved in type 2 inflammatory immune responses, secreted by M2 macrophages that have been shown to play a key role in supporting fibrosis development and progression (24–27). CCL24 is also a potent chemotactic ligand that drives immune cell recruitment, following tissue damage, infection, or injury (28, 29). In liver biopsies from patients with nonalcoholic steatohepatitis (NASH) and hepatocellular carcinoma, both CCR3 and CCL24 were found to be hyperexpressed, and high CCR3 expression in hepatic fibroblasts was demonstrated in both liver diseases (30, 31). We have also previously demonstrated that CCL24-knockout mice, which do not exhibit any overt phenotype under normal conditions, have attenuated fibrosis development and inflammation in a NASH-induced liver injury model (29). Additionally, in several animal models of NASH and liver fibrosis, CCL24 blockage by the anti-CCL24 monoclonal antibody, CM-101 (D8), significantly reduces liver damage, most importantly by directly reducing liver fibrosis (30).

Considering the dual role of CCL24 in induction of pro-fibrotic immune response, as well as direct liver and skin fibroblast activation, we set out to establish the relevance of CCL24 in the pathophysiology of PSC and its specific role in fibrosis progression in this disease.

## Results

### CCL24 is expressed and secreted from mouse liver macrophages in a PSC experimental model.

Macrophages, the most abundant liver immune cells, play a key role in cholestatic diseases, with a predominant role in mediating inflammation, cholangitis, and fibrosis (32–34). Hepatic macrophages consist of 2 main populations: resident macrophages and monocyte-derived macrophages (35–37). The macrophages can be further polarized and drive processes that support acute and chronic inflammation, fibrosis, and cancer (27, 32–34).

Multidrug resistance gene 2–knockout (Mdr2^–/–^) mice are considered the gold standard animal model for sclerosing cholangitis. These animals develop features that are very similar to PSC in terms of cholangitis, severe ductular reaction, and fibrosis (38).

**Figure 1:**
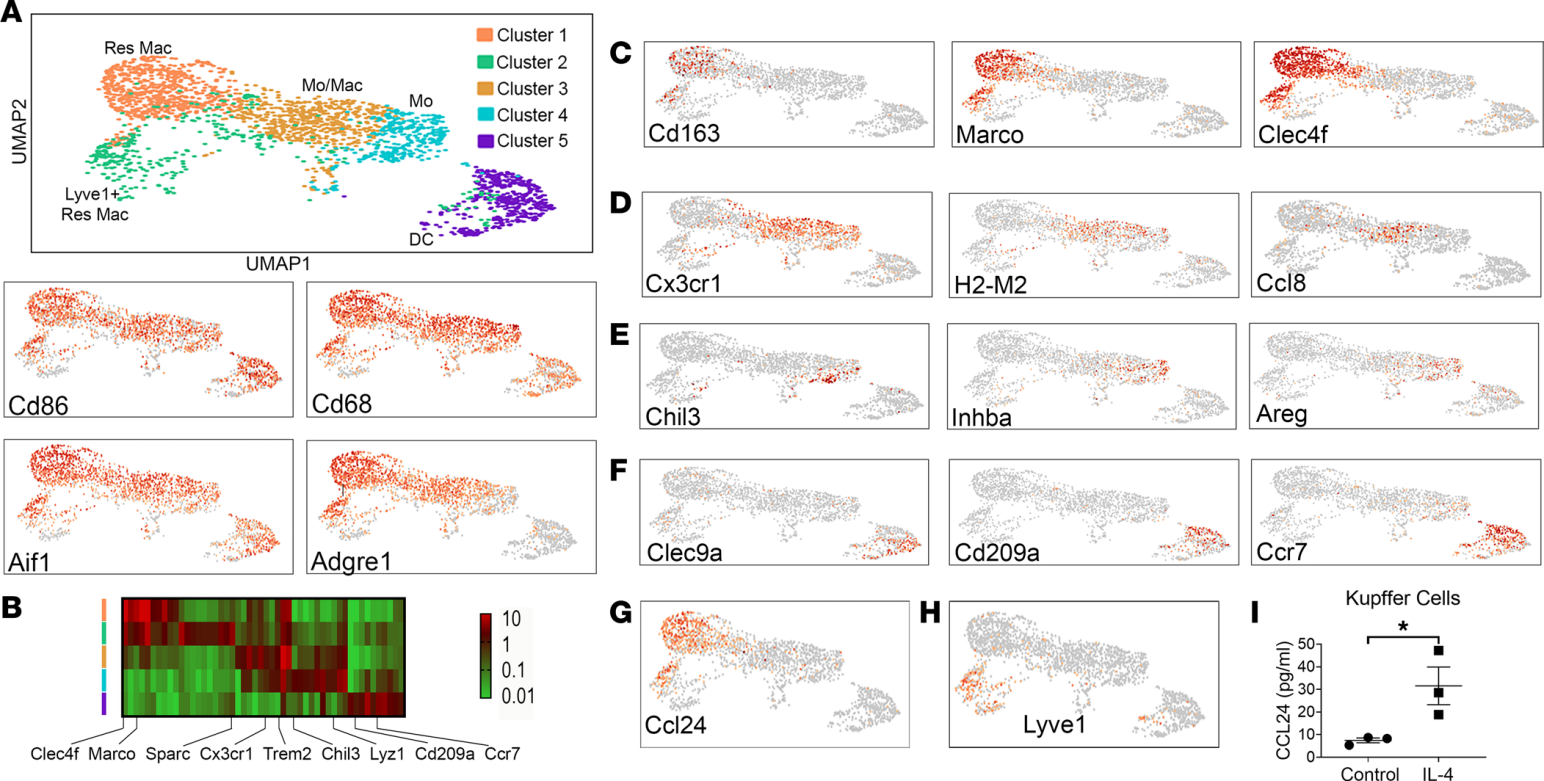
scRNA-Seq of monocytes and macrophages from 3-month-old Mdr2^–/–^ mice. (**A**) Five populations of monocytes/macrophages and dendritic cells (DCs) were identified using general mononuclear phagocyte markers: Cd86, Aif1 (Iba1), Cd68, and Adgre1 (F4/80). (**B**) Heatmap, mononuclear phagocyte cluster marker genes (left, color coded by cluster), exemplar genes labeled (bottom). Genes columns, clusters rows. (**C**–**H**) Each population was characterized using specific genes. Res Mac express Cd163, Marco, and Clec4f (**C**). Mo/Mac identified by Cx3cr1, H2-M2, and Ccl8 (Mcp2) (**D**). Mo were characterized by Chil3, Inhba, and Areg expression (**E**). DCs were characterized by Clec9a, Cd209a (DC-SIGN), and Ccr7 (**F**). (**G**) Ccl24’s robust expression by liver-resident macrophages. (**H**) A potentially unique subpopulation of macrophages express Ccl24 as well as endothelial cell gene markers like Lyve1. (**I**) Secreted levels of CCL24 from isolated mouse Kupffer cells that were cultured with or without IL-4 supplement (20 ng/mL). Data are shown as mean ± SEM (*n* = 3). *P ≤ 0.05, *t* test. Res Mac, resident macrophages; Mo, monocytes; Mo/Mac, monocyte-derived macrophages; Lyve1, lymphatic vessel endothelial hyaluronan receptor 1.

Based on the predominant role of macrophages in the pathogenesis of PSC and the known expression and involvement of CCL24 in macrophages, we aimed to test the expression pattern of CCL24 in different macrophage populations using the experimental Mdr2^–/–^ mice. For this purpose, we utilized the single-cell RNA-sequencing (scRNA-Seq) method using 3-month-old Mdr2^–/–^ mice. We first extracted cell populations using general mononuclear phagocyte markers: Cd68, adhesion G protein–coupled receptor E1 (Adgre1; F4/80), Cd86, integrin alpha M (Itgam; Cd11b), allograft inflammatory factor 1 (Aif1; ionized calcium-binding adapter molecule 1, Iba1), and membrane-spanning 4-domains, subfamily A, member 7 (Ms4a7). Clustering of the mononuclear phagocytes identified 5 clusters (Figure 1, A and B), classified as resident liver macrophages, monocyte-derived macrophages, tissue monocytes, and dendritic cells (DCs). Two clusters of resident liver macrophages (Kupffer cells, clusters 1 and 2) were identified by Cd163, macrophage receptor with collagenous structure (Marco), and C-type lectin domain family 4, member f (Clec4f) (Figure 1C and Supplemental Table 1; supplemental material available online with this article; https://doi.org/10.1172/jci.insight.162270DS1). Monocytes (cluster 4) and monocyte-derived macrophages (cluster 3), which are usually recruited to injured sites, expressed Cx3cr1 and histocompatibility 2, M region locus 2 (H2-M2). The monocyte-derived macrophages highly expressed Ccl8 (Mcp2), whereas the monocytes that were not differentiated into macrophages expressed chitinase-like 3 (Chil3), inhibin beta-A (Inhba), and amphiregulin (Areg) (Figure 1, D, and E, and Supplemental Table 1). The DC cluster was composed of both Clec9a-expressing classical type 1 DCs and Cd209a-expressing classical type 2 DCs (Figure 1F and Supplemental Table 1). Interestingly, we found that in Mdr2^–/–^ mouse livers, CCL24 was highly expressed by resident macrophages (Figure 1G). A further finding among the CCL24-expressing macrophages was a potentially unique subpopulation of resident macrophages that expressed Lyve1 (cluster 2, Figure 1H). To corroborate these results, we isolated mouse Kupffer cells and measured CCL24 secretion. We indeed observed CCL24 secretion from these cells, which was increased with addition of IL-4, a cytokine that induces type 2 immune responses (Figure 1I). Taken together, the high expression of CCL24 observed in macrophages in experimental Mdr2^–/–^ mice, which was similar to the expression expected in patients with PSC, supports the use of the Mdr2^–/–^ mouse model for studying the role of CCL24 in PSC.

### CCL24 inhibition by CM-101 (D8) results in reduced mouse liver macrophage accumulation and decreased bile mass in the Mdr2^–/–^ mouse model.

CM-101 (D8), a monoclonal antibody that targets and neutralizes CCL24 signaling, was used to evaluate the effect of blocking CCL24 on the development of liver cholestasis and fibrotic injury in the Mdr2^–/–^ mouse model. Mice were treated (5 and 10 mg/kg) twice weekly for 6 weeks (from age 6 to 12 weeks), by subcutaneous injection of the antibody or PBS vehicle control.

**Figure 2:**
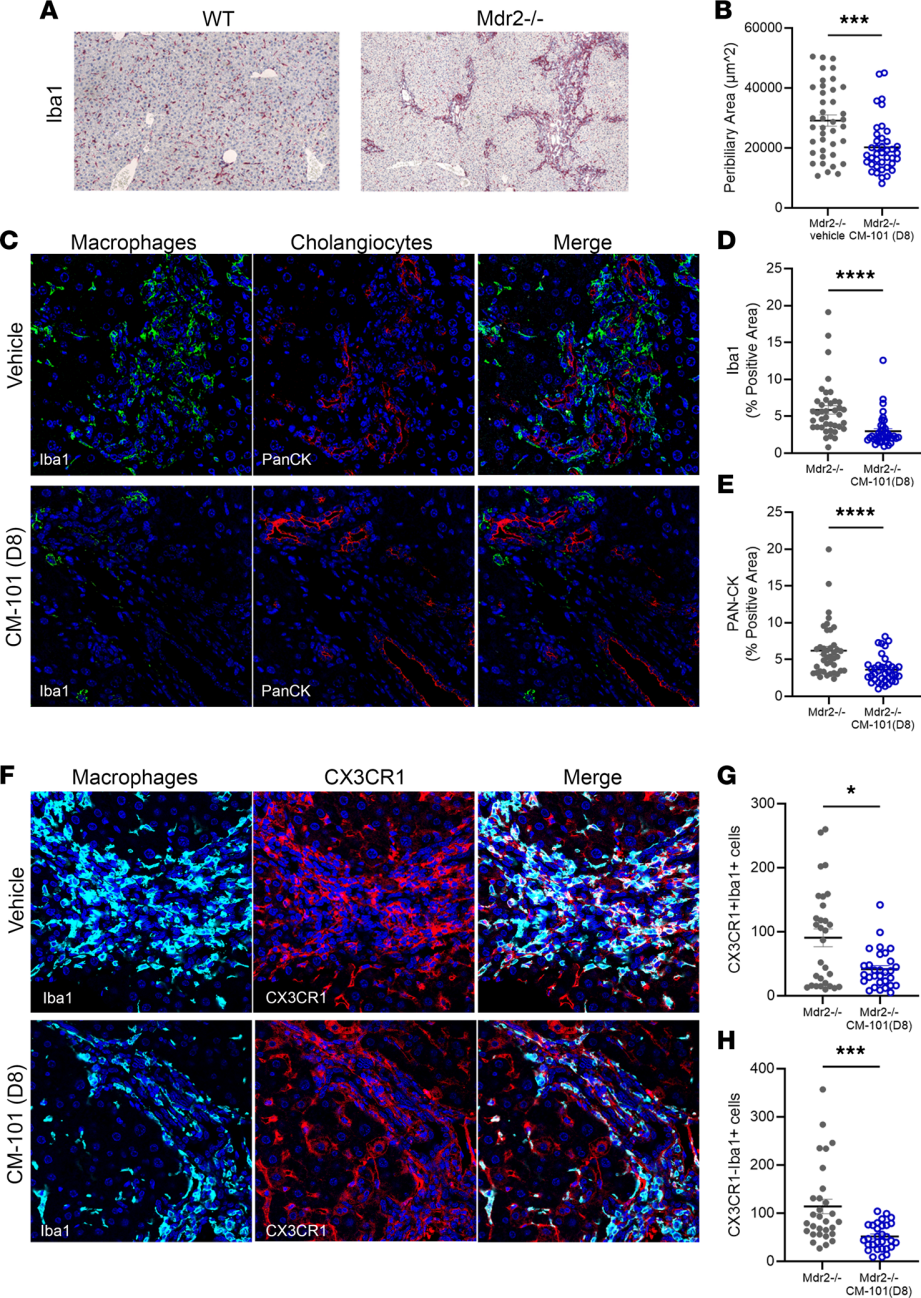
CM-101 (D8) abrogates macrophage accumulation and reduces ductular expansion in Mdr2^–/–^ mice. (**A**) Immunohistochemistry staining of Iba1 in 3-month-old WT and Mdr2^–/–^ mice. (**B**–**E**) Immunofluorescence staining of liver cholangiocytes and macrophages in CM-101–treated (10 mg/kg) or vehicle-treated Mdr2^–/–^ mice. (**B**) Quantification of the injured peribiliary area, based on Iba1 and pan-CK staining (*n* = 50 fields, 5 mice in each group). (**C**) Representative Mdr2^–/–^ liver sections stained against Iba1 for macrophages and pan-CK for cholangiocytes (×40 original magnification). (**D** and **E**) Quantification of Iba1 and pan-CK staining (*n* = 38–40 fields, 7 mice in each group). (**F**) Representative Mdr2^–/–^ liver sections stained against Iba1 and CX3CR1 for recruited macrophages (×40 original magnification). (**G** and **H**) Quantification of recruited macrophages (Iba1-positive and CX3CR1-positive, **G**) and nonrecruited macrophages (Iba1-positive and CX3CR1-negative, **H**) (*n* = 30 fields, 5 mice in each group). Data are mean ± SEM. Mann-Whitney test, *P ≤ 0.05, ***P ≤ 0.001, ****P ≤ 0.0001.

Staining of liver sections using Iba1 marker that stains both resident liver macrophages and monocyte-derived macrophages demonstrated massive periductal macrophage accumulation surrounding cholangiocytes (Figure 2, A and C). Blocking CCL24 using 10 mg/kg CM-101 (D8) treatment reduced this inflammatory process with a 2-fold decrease in macrophage staining (Figure 2D) and a general decrease in the area of peribiliary inflammation (Figure 2B). To characterize which macrophage populations were affected by CCL24 blockade, we labeled liver sections with Iba1 and CX3CR1, which marks recruited macrophages (Figure 2F). We observed a 3-fold reduction in recruited macrophages (Figure 2G), as well as a 2-fold reduction in CX3CR1-negative macrophages (Figure 2H), suggesting that resident macrophages were also affected.

To further assess whether the reduced macrophage presence in 10 mg/kg CM-101–treated mice is accompanied by reduced cholangiocyte proliferation, we stained with pan-cytokeratin (pan-CK) for identification of cholangiocytes, the major cell subpopulation that accumulates in the ductular reaction of Mdr2^–/–^ mice (39). Following treatment with CM-101 (D8), pan-CK staining was reduced by 40%, suggesting that CCL24 blockade attenuates ductular expansion, compared with extensive bile proliferation and expansion in vehicle-treated controls (Figure 2, C and E).

### Blocking CCL24 signaling reduces mouse liver injury and fibrosis.

**Figure 3:**
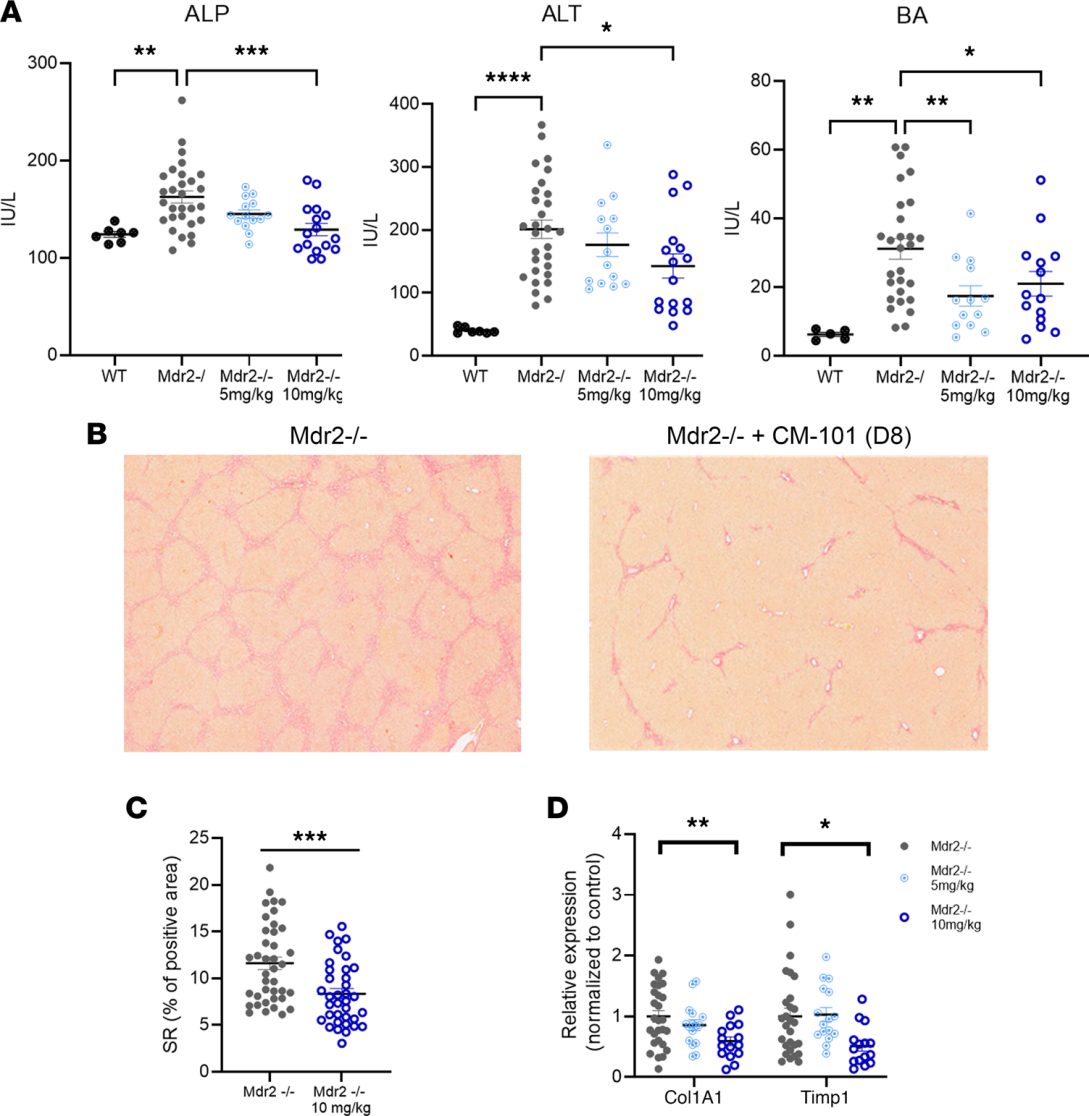
Mdr2^–/–^ mice treated with CM-101 (D8) exhibit reduced liver damage and fibrosis. (**A**) Serum levels of liver enzymes ALP and ALT and BA (*n* = 7, 29, 14, and 16 for WT, Mdr2^–/–^+vehicle, Mdr2^–/–^+5 mg/kg CM-101, and Mdr2^–/–^+10 mg/kg CM-101, respectively). (**B** and **C**) Sirius red (SR) staining in Mdr2^–/–^ mice. Representative images (**B**) and quantification of SR-stained area (**C**) (*n* = 12 and 13 for vehicle and 10 mg/kg, respectively. 3 fields per mouse). (**D**) Timp1 and Col1a1 expression was measured by real-time PCR in livers of treated and vehicle-treated Mdr2^–/–^ mice (*n* = 28, 16, and 15 mice for vehicle, 5 mg/kg, and 10 mg/kg, respectively). Data are mean ± SEM. *P ≤ 0.05, **P ≤ 0.01, ***P ≤ 0.001, ****P ≤ 0.0001, ANOVA followed by Holm-Šídák multiple comparisons test.

Next, we tested the effect of CM-101 (D8) on biomarkers of liver inflammation, fibrosis, and cholestasis. Liver biochemistry parameters, alkaline phosphatase (ALP), alanine aminotransferase (ALT), and bile acid (BA), were all elevated in Mdr2^–/–^ mice compared with control WT mice (Figure 3A). CM-101 (D8) treatment resulted in a dose-dependent reduction of serum ALP after 6 weeks of treatment, compared with Mdr2^–/–^ controls, with levels similar to those seen in WT animals (162 ± 34, 145 ± 16.1, and 130 ± 25 IU/L for Mdr2^–/–^ control, 5 mg/kg CM-101, and 10 mg/kg CM-101, respectively). ALT was also reduced in the treated animals, with a significant decrease in the group treated with 10 mg/kg (143 ± 77.1 IU/L) compared with vehicle control (201 ± 79.2 IU/L), while the 5 mg/kg dose (176 ± 70.0 IU/L) did not reach significance (Figure 3A). A significant reduction of serum BA levels was seen in mice treated with both doses of CM-101 (D8) compared with Mdr2^–/–^ controls, indicating reduced cholestatic injury (31.18 ± 15.8, 17.44 ± 10.5, and 21.8 ± 13 IU/L for Mdr2^–/–^ control, 5 mg/kg CM-101, and 10 mg/kg CM-101, respectively). No significant change in bilirubin was detected.

To specifically evaluate the antifibrotic effects of blocking CCL24 in this model, we assessed fibrosis severity and quantified liver collagen content using Sirius red (SR) staining. Treating Mdr2^–/–^ animals with CM-101 (D8) resulted in a reduction of liver fibrosis, which was moderate in the animals treated with the 5 mg/kg dose (data not shown) and significant in animals treated with the 10 mg/kg dose of CM-101 (D8), with a 30% decrease in SR staining and reduction in bridging fibrosis (Figure 3, B and C). Supporting the histological fibrosis assessment, mRNA levels of pro-fibrotic genes, collagen, type I, alpha 1 (Col1a1), and tissue inhibitor of metalloproteinase 1 (Timp1), were significantly reduced by 40% and 50%, respectively, in the livers of animals treated with 10 mg/kg CM-101 (D8) compared with vehicle-treated controls (Figure 3D).

To summarize the observed changes in liver and serum in relation to disease severity, we evaluated the correlation between liver fibrosis (SR), liver macrophage accumulation (Iba1), and cholangiocyte proliferation (pan-CK). We observed high correlations between all markers (Supplemental Figure 1, A and B), where the highest correlation was observed between pan-CK and Iba1 (*r* = 0.91, *P* < 0.0001).

### CCL24 neutralization reduces senescence, proliferation, and ECM remodeling pathways in mouse peribiliary cells.

**Figure 4:**
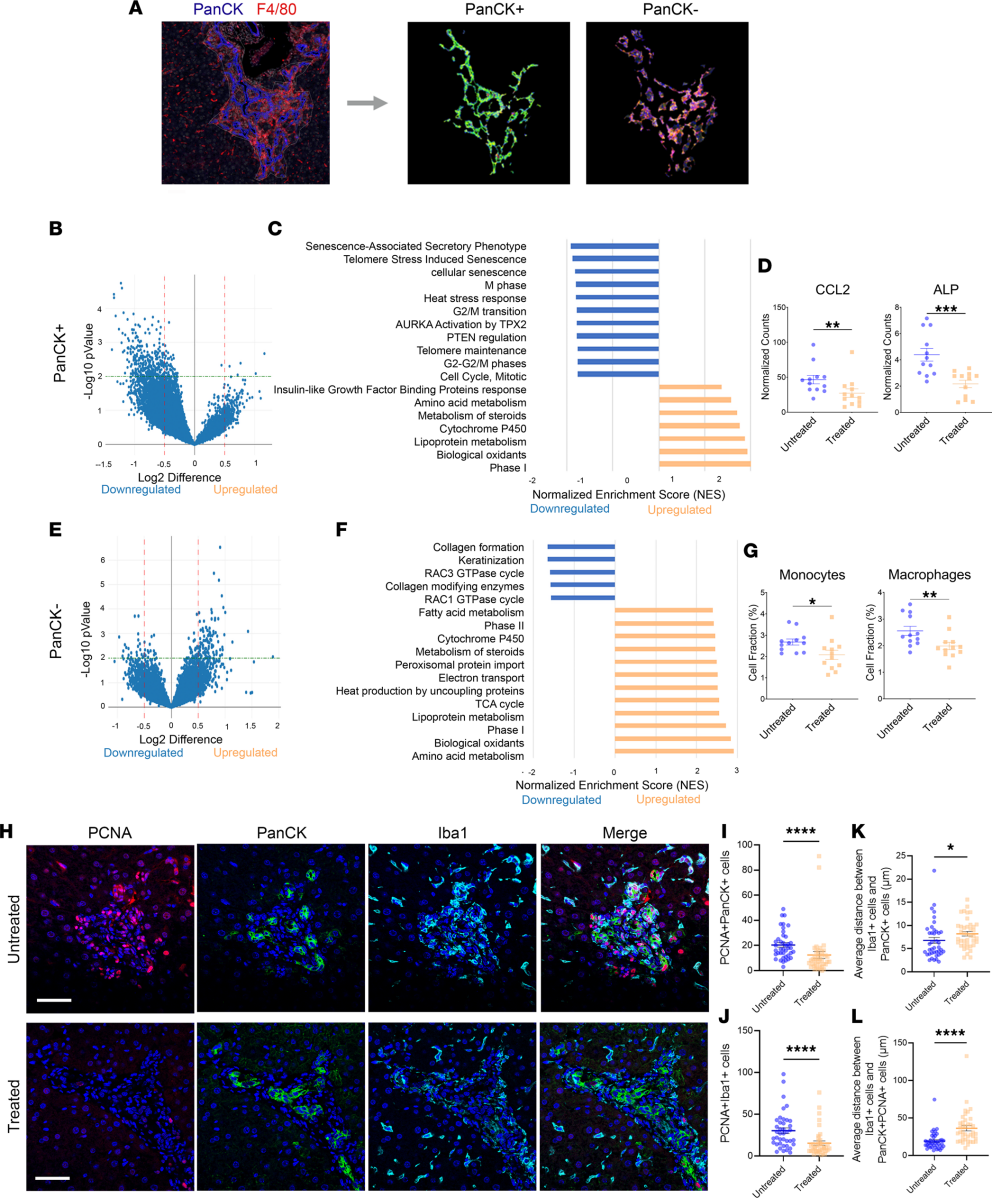
CM-101 (D8) treatment alters cholangiocyte state and immune cell composition in the peribiliary area in Mdr2^–/–^ mice. (**A**–**G**) Spatial gene expression analysis (NanoString GeoMx technology) was performed in peribiliary areas from CM101–treated (10 mg/kg) or vehicle-treated Mdr2^–/–^ mice. (**A**) Regions of interest (ROIs) were separated to pan-CK–positive area or pan-CK–negative area. (**B** and **E**) Volcano plot showing the differential gene expression. (**C** and **F**) Waterfall plot of gene set enrichment analysis signatures ranked by normalized enrichment score (NES). (**D**) CCL2 and alkaline phosphatase liver/bone/kidney isozyme gene expression analysis in pan-CK–positive peribiliary areas of treated and vehicle-treated Mdr2^–/–^ mice (*n* = 12; 4 mice, 3 ROIs for each tissue). (**G**) Monocytes and macrophages’ percentages in pan-CK–negative peribiliary areas. Bulk RNA counts were deconvoluted using ImmGen cell signatures (*n* = 12; 4 mice, 3 ROIs for each tissue). (**H**–**L**) Proliferating macrophages and cholangiocytes were analyzed by immunofluorescence staining against proliferating cell nuclear antigen (PCNA), pan-CK, and Iba1. (**H**) Representative Mdr2^–/–^ liver sections of CM-101– or vehicle-treated animals (×40 original magnification). (**I** and **J**) Quantification of proliferating cholangiocytes (**I**) and macrophages (**J**) by total number in peribiliary area (*n* = 40; 5 mice). (**K** and **L**) Proximity analysis between macrophages and either total cholangiocytes (**K**) or proliferating cholangiocytes (**L**) (*n* = 40; 5 mice). Mann-Whitney test, *P ≤ 0.05, **P ≤ 0.01, ***P ≤ 0.001. Scale bar represents 50 μm.

Owing to the known patchy disease distribution in PSC, with unevenly distributed peribiliary damaged areas, which are surrounded by healthy liver tissue, we sought to characterize the spatial transcriptomics alterations in the biliary areas following CCL24 blockade. To study the effect mediated by inhibition of CCL24, we used the GeoMx platform followed by whole-transcriptome sequencing (NanoString). Livers from Mdr2^–/–^ mice treated for 6 weeks with 10 mg/kg CM-101 (D8) or vehicle were stained by pan-CK, CD45, F4/80, and nuclear staining. This staining allowed us to define a region of interest enriched in bile ducts and the surrounding inflammatory cells. The tissue was further segmented into pan-CK–positive and pan-CK–negative samples, which enabled cholangiocytes and noncholangiocytes’ fractionation (Figure 4A). Each segment went through whole-transcriptome sequencing. Interestingly, we found that while treatment with CM-101 (D8) led mostly to reduced gene expression in the cholangiocytes (Figure 4B), genes from pan-CK–negative populations (representing the immune cells) were mainly upregulated (Figure 4E). Gene set enrichment analysis of pan-CK–positive differentially expressed genes showed reduction in cellular pathways related to cell senescence and proliferation in cholangiocytes (Figure 4C). Furthermore, immunostaining for senescence (Supplemental Figure 1C) and proliferation markers (Figure 4, H–L) corroborated that blocking CCL24 affected both processes. Specifically, the ALP gene was downregulated following CM-101 (D8) treatment, highlighting that the reduction seen in the serum ALP of treated mice directly reflects its reduced expression in cholangiocytes. Additionally, the inhibition of CCL24 downregulated CCL2 expression in cholangiocytes (Figure 4D), suggesting a reduced inflammatory status of the epithelial cells. In the pan-CK–negative cell population, we identified reduction of pathways related to ECM remodeling and upregulation of pathways related to metabolic activity (Figure 4F). Finally, using cell deconvolution, we assessed the percentage of different immune cell populations in the pan-CK–negative population (Figure 4G). Treatment with CM-101 (D8) showed a reduction in the percentage of macrophages and monocytes in the peribiliary area, suggesting a reduction in recruitment of these immune populations into the injured peribiliary area.

We further characterized the effect of CCL24 on proliferating cells in the injured peribiliary area, by staining PCNA (Figure 4H). We observed a robust reduction in proliferating cells in CM-101–treated mice. Using Iba1 and pan-CK as markers for macrophages and cholangiocytes, respectively, we saw that both of these proliferating populations decreased following CCL24 blockade (Figure 4, H–J). To determine whether the macrophages accumulated around proliferating bile ducts, we used machine learning analysis to calculate the average distances between cells. We analyzed the proximity between macrophages and proliferating cholangiocytes (Figure 4L) or between macrophages and total cholangiocytes (Figure 4K). Macrophages accumulated around both cholangiocyte populations, with higher proximity to nonproliferating cholangiocytes. Treatment with CM-101 (D8) interfered with macrophage accumulation around cholangiocytes, leading to higher distances between these cells.

Finally, we corroborated the effect of blocking CCL24 in an additional cholangitis model. We evaluated cholestatic fibrosis induced by bile duct ligation in rats (Supplemental Figure 2, A–C). Blocking CCL24 with i.v. twice-weekly injection of 10 mg/kg CM-101 (D8) reduced biliary damage, as observed by reduced cholangiocyte proliferation (pan-CK staining) and reduced liver fibrosis (SR staining).

### CCL24 is elevated by human macrophages and cholangiocytes under pro-fibrotic environmental conditions.

**Figure 5:**
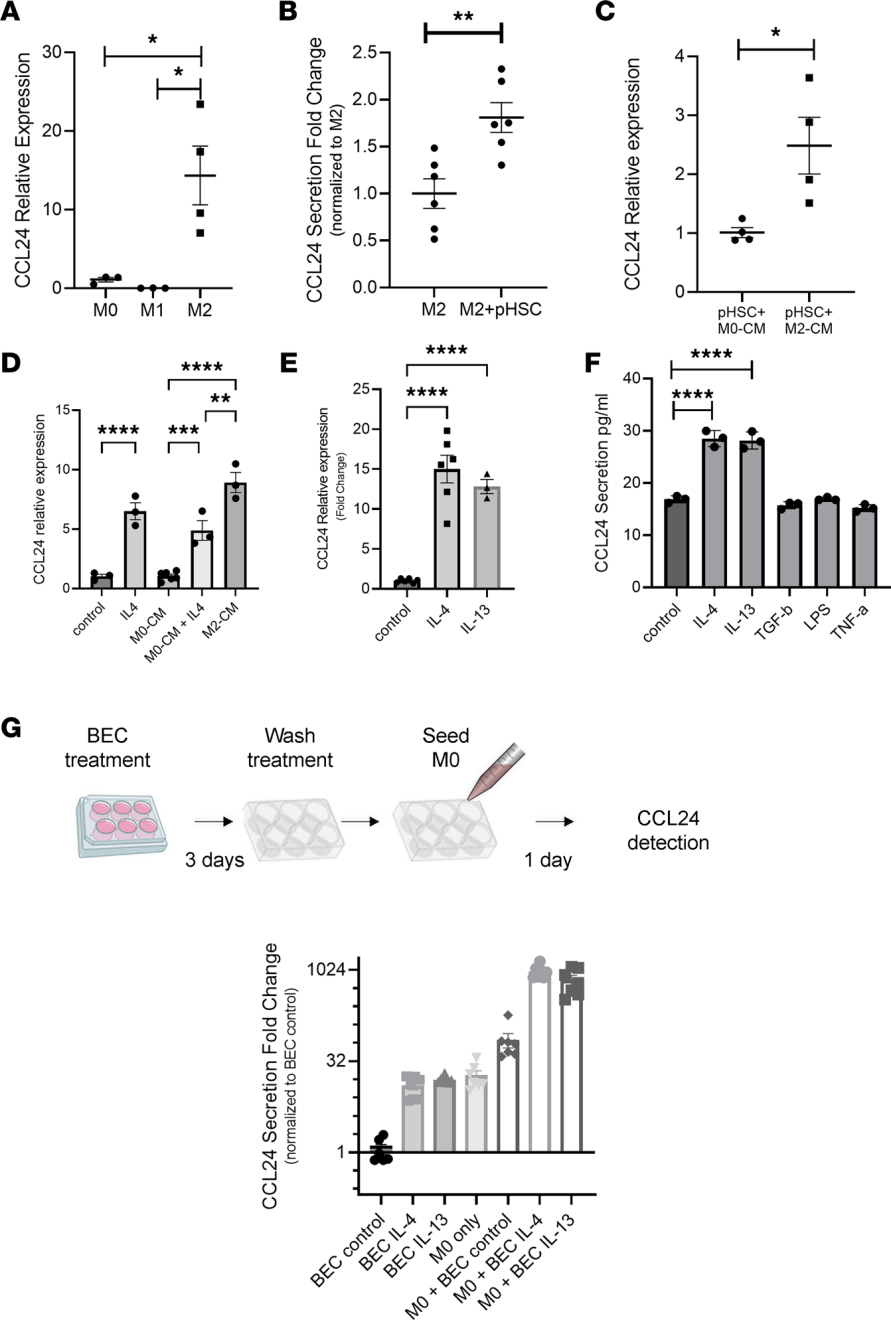
CCL24 is upregulated under pro-fibrotic conditions in human macrophages, hepatic stellate cells, and cholangiocytes. (**A**) M2 macrophages upregulate CCL24 gene expression (*n* = 3–4). (**B**) Secreted levels of CCL24 expression measured in M2 macrophages incubated with or without HSCs (*n* = 6). (**C**) Gene expression of CCL24 in HSCs cultured with M0 or M2 macrophage conditioned medium (M0-CM and M2-CM, respectively) (*n* = 4). (**D**) Gene expression of CCL24 in LX2 cells cultured with starvation medium or M0 or M2 macrophage conditioned medium (M0-CM and M2-CM, respectively). IL-4 was supplemented at 20 ng/mL (*n* = 3). (**E**) CCL24 gene expression in primary cholangiocytes following treatment with IL-4 or IL-13 (*n* = 3–6). (**F**) CCL24 quantified by ELISA following different treatments of primary cholangiocytes (*n* = 3). (**G**) Secreted levels of CCL24 in macrophages (M0) cocultured with cholangiocytes that had been pretreated with vehicle, IL-4 or IL-13. Upper scheme, experiment procedure: cholangiocytes were treated for 3 days with vehicle, IL-4 or IL-13, then washed, and M0 cells were added on top of the cholangiocytes for 24 hours. Lower graph, detection by ELISA (*n* = 7). All data are presented as mean ± SEM. *P ≤ 0.05, **P ≤ 0.01, ***P ≤ 0.001, ****P ≤ 0.0001, *t* test (**A**–**C**) or ANOVA followed by Holm-Šídák multiple comparisons test (**D**–**F**).

Based on the extensive expression of CCL24 seen in mouse macrophages and the significant attenuation of inflammation following CM-101 treatment via reduced monocyte/macrophage accumulation in the livers of Mdr2^–/–^ mice, we aimed to assess CCL24 expression in human macrophages. M2 macrophages are important promotors of the fibrotic niche, as they maintain an immune environment enriched by pro-fibrotic factors. CCL24 is a known marker of M2 polarization and is upregulated in vitro in monocyte-derived macrophages cultured with IL-4 (40). Macrophage differentiation of freshly isolated peripheral monocytes showed that M2 polarization increased CCL24 gene expression (Figure 5A) that was also reflected by high levels of CCL24 secretion compared with control M0 unpolarized cells (data not shown), resembling the CCL24 upregulation of IL-4–stimulated Kupffer cells (Figure 1I).

**Figure 6:**
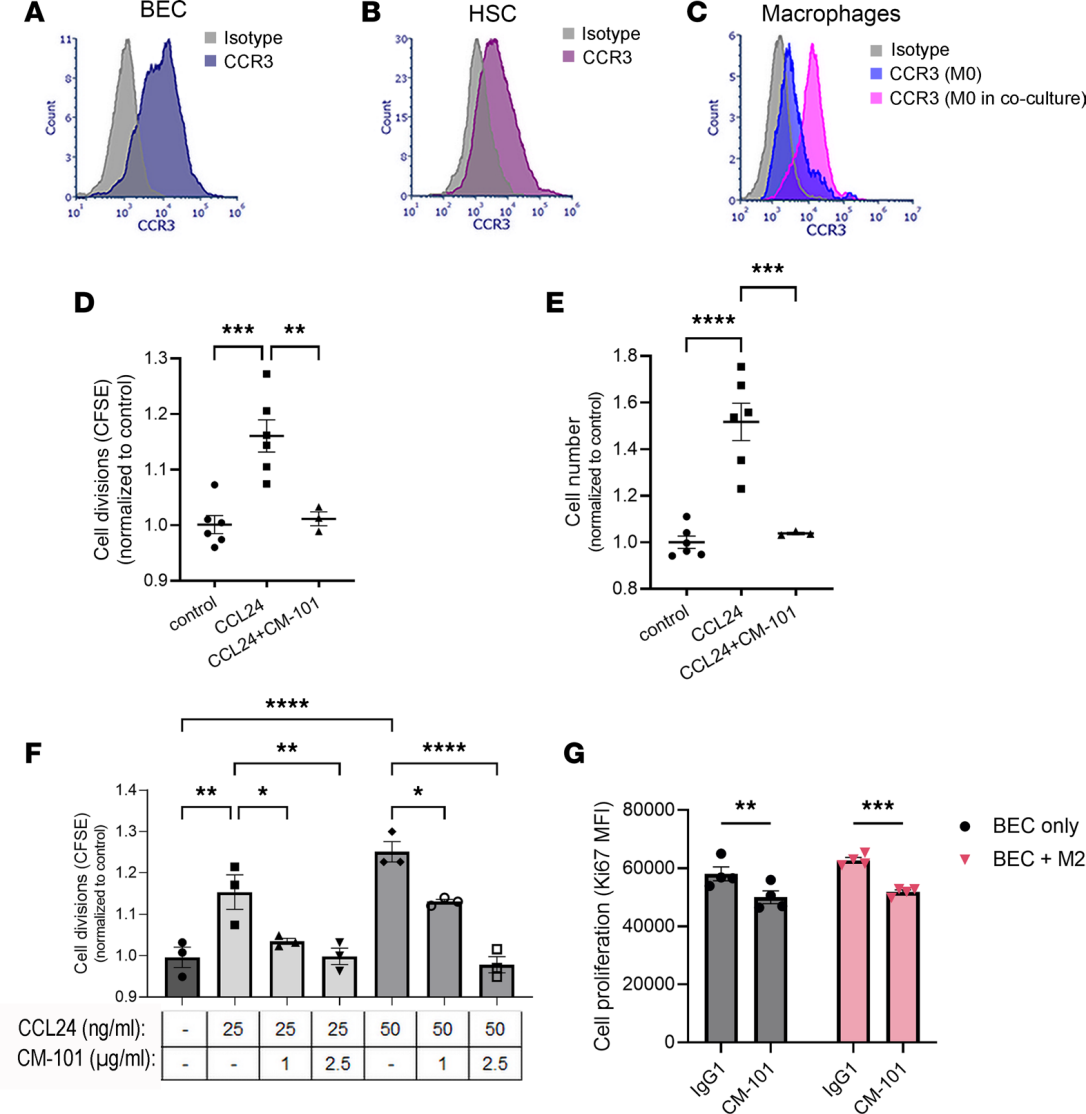
CCL24 induces human HSC and cholangiocyte proliferation and macrophage polarization. (**A**–**C**) Macrophages, HSCs, and cholangiocytes express the CCR3 receptor. Representative histograms of flow cytometric analysis of surface CCR3 expression in primary cholangiocytes (**A**), primary HSCs (**B**), and monocyte-derived macrophages (**C**). The experiment was conducted 4 times. Macrophages in coculture are gated based on expression of CCR5 and CD206. (**D**–**F**) Proliferation of CFSE-labeled HSCs (**D** and **E**) or LX2 cells (**F**) with or without CM-101 (*n* = 3–6). (**G**) Proliferation of BECs with IgG1 or CM-101 was measured based on Ki-67 expression. Cholangiocytes were incubated with or without M2 macrophages with 5 μg/mL of CM-101 or IgG1 isotype control (*n* = 4). Data are mean ± SEM. *P ≤ 0.05, **P ≤ 0.01, ***P ≤ 0.001, ****P ≤ 0.0001, ANOVA followed by Holm-Šídák multiple comparisons test.

Primary hepatic stellate cells (HSCs), liver-specific fibroblasts known to play a central role in liver fibrogenesis, showed CCR3 expression when analyzed by flow cytometry (Figure 6B). Coculturing pro-fibrotic polarized M2 macrophages with human primary HSCs resulted in even higher levels of CCL24 secretion compared with M2 macrophages alone, revealed by a 70% increase in CCL24 concentration in cell media (Figure 5B). Under normal culturing conditions, HSCs neither produced nor secreted CCL24 in vitro, as indicated by undetectable levels of CCL24 (data not shown). We therefore set out to investigate whether the increased CCL24 levels observed in the coculture of these 2 cells is attributed to elevated production in M2 macrophages or HSCs. Incubation of HSCs with conditional medium from M2 (M2-CM) induced a 2-fold increase in CCL24 gene expression compared with culturing with conditioned medium from unpolarized M0 macrophages (M0-CM, Figure 5C). These findings were corroborated using the LX2 HSC line. LX2 cells stimulated with either IL-4 or M2 conditioned medium upregulated CCL24 expression by 6- or 9-fold, respectively (Figure 5D).

To better understand the specific regulation of CCL24 under pro-fibrotic conditions relevant to PSC, we additionally evaluated CCL24 expression in cultured intrahepatic cholangiocytes (biliary epithelial cells, BECs). Culturing these cells in the presence of several pro-fibrotic and pro-inflammatory cytokines and evaluating CCL24 expression, we found that IL-4 and IL-13 induced a substantial increase in CCL24 gene expression, compared with untreated controls (Figure 5E). Secretion of CCL24 into culture media was also significantly increased in IL-4– and IL-13–treated cholangiocytes (Figure 5F), indicating that this chemokine is upregulated in immune cells, fibroblasts, and epithelial cells in a pro-fibrotic cytokine environment. Next, we cocultured unpolarized M0 cells with pro-fibrotic cholangiocytes (i.e., pretreated with IL-4 or IL-13) and analyzed CCL24 secretion. We observed a synergic effect of CCL24 secretion (Figure 5G), whereas the coculture of M0 with IL-4–pretreated cholangiocytes had more than 20-fold higher secretion compared with the summation of the two.

### CCL24 induces human cholangiocyte and HSC proliferation in vitro.

The extensive expression of CCL24 by immune cells and cholangiocytes induced by pro-fibrotic IL-4/IL-13 cytokines suggests that under pro-fibrotic, pro-inflammatory conditions, the periductal space may be saturated with secreted CCL24 that can activate the CCR3-positive cells surrounding this area. We therefore turned to examine CCR3 expression on cholangiocytes, HSCs, and macrophages. We documented CCR3 expression in these primary cells (Figure 6, A–C). Moreover, when incubated with cholangiocytes, macrophages upregulated CCR3 expression.

We previously published that CCL24 can directly activate HSCs, inducing transition to myofibroblasts with increased α–smooth muscle actin (α-SMA) expression and enhanced cell motility (30). In addition to activation, fibroblast proliferation is an important factor in the establishment and propagation of fibrosis. Incubation of HSCs with CCL24 significantly increased proliferation, evidenced by a 50% increase in cell counts (Figure 6E) and a proportionate reduction in CFSE staining (Figure 6D). CM-101, a monoclonal antibody that specifically blocks CCL24, inhibited the HSC proliferation and reduced cell count back to baseline levels. Supporting these results, culturing LX2 cells with increasing levels of CCL24 resulted in a dose-dependent increase in proliferation with a 25% increase in proliferation index that was blocked by CM-101 at all tested doses (Figure 6F).

As the in vivo models suggested that blocking CCL24 can inhibit cholangiocyte proliferation, we examined the in vitro proliferation of primary cholangiocytes when grown alone or in coculture with macrophages (Figure 6G). Blocking CCL24 with CM-101 reduced cholangiocyte proliferation, as evidenced by CFSE staining (data not shown) and Ki-67 staining (Figure 6G). Together with the observation of reduced proliferation in liver tissue of CM-101–treated Mdr2^–/–^ mice, these results suggest that CCL24 can directly induce proliferation of cholangiocytes.

Finally, because macrophages upregulated CCR3 when cocultured with cholangiocytes (Figure 6C), we assessed whether unpolarized M0 macrophages shift toward a pro-fibrotic polarization state when incubated with CCL24-expressing cholangiocytes. We examined changes in polarization markers on M0 macrophages that were cocultured with cholangiocytes pretreated with IL-4 or IL-13 (Supplemental Figure 3). We observed changes in CD16, CD86, CD206, triggering receptor expressed on myeloid cells 2 (Trem2), and CCR5, with the most prominent changes were in CD206 and Trem2, which are associated with a pro-fibrotic IL-4–induced polarization. These coculture experiments shed light on the aggravation of the disease within the tissue. In the injured peribiliary area, macrophages and cholangiocytes interact to stimulate each other toward a pro-fibrotic state.

### Expression of CCL24 and its receptor CCR3 in liver biopsies, immune cells, and serum of human patients with PSC.

**Figure 7:**
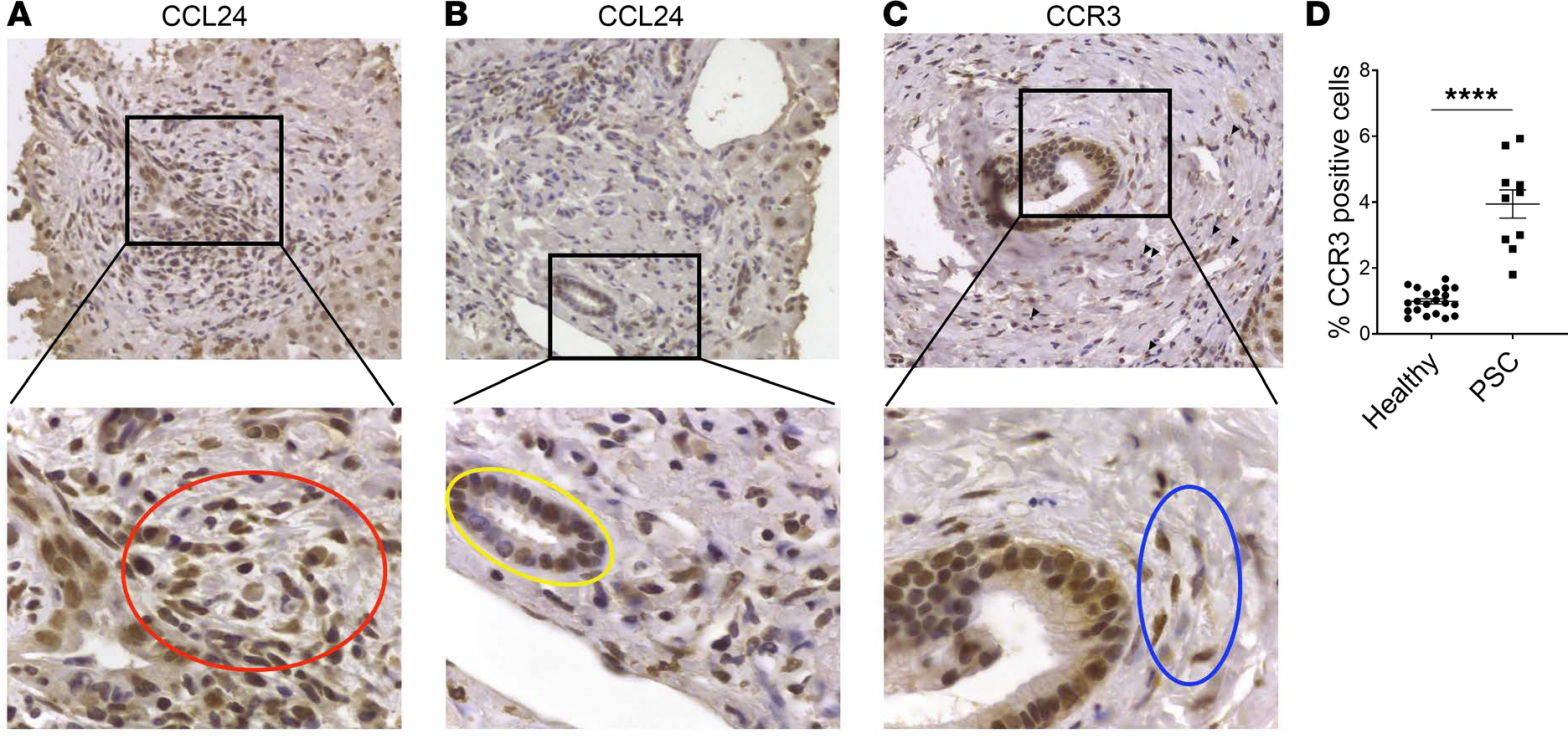
CCL24 and CCR3 are highly expressed in the liver and on PBMCs of patients with PSC and correlate with fibrotic biomarkers. (**A** and **B**) CCL24 staining in PSC sections (×40 original magnification). Positive staining is detected in inflammatory mononuclear cells (red circle) and cholangiocytes (yellow circle). (**C**) CCR3 staining (×40 original magnification) observed in inflammatory cells (black arrowheads) or fibroblasts (blue circle) surrounding the bile duct. (**D**) CCR3 staining of PBMCs from PSC patients (*n* = 10) and healthy individuals (*n* = 22). Data are mean ± SEM. ****P ≤ 0.0001, *t* test.

To study the expression and localization of CCL24 in the livers of patients with PSC, we evaluated the pattern of CCL24 expression in liver biopsies from patients with PSC by immunohistochemistry, specifically focusing on the damaged periductal space. CCL24 was markedly upregulated in biopsies taken from patients with PSC and was mainly expressed by inflammatory cells surrounding the bile ducts. Due to the substantial inflammatory insult in PSC, reflected by massive accumulation of resident and recruited immune cells in the periductal space, CCL24-positive staining in this area was extensive. Similar to the cellular expression demonstrated in vitro, we found great CCL24 staining in cholangiocytes, which play a central role in the pathophysiology of PSC (Figure 7, A and B).

We also evaluated the expression of CCR3 by immunohistochemistry. Interestingly, specific CCR3 expression was evident in cholangiocytes and in surrounding fibroblasts. CCR3 was also stained in mononuclear cells within the inflammatory foci, which colocalized with bile ducts (Figure 7C). These results show that CCL24 and CCR3 are coexpressed in the periductal area and in cells relevant to the tissue damage seen in patients with PSC.

In addition to evaluating hepatic CCR3 expression, we explored whether patients with PSC exhibit high CCR3 expression levels on isolated circulating PBMCs. Flow cytometry analysis showed higher CCR3 expression in PBMCs of PSC patient samples (3.94% ± 1.35%) compared with healthy donor samples (0.98% ± 0.36%), with a 3.5-fold increase in the percentage of CCR3-positive cells found in the circulation (Figure 7D).

**Figure 8:**
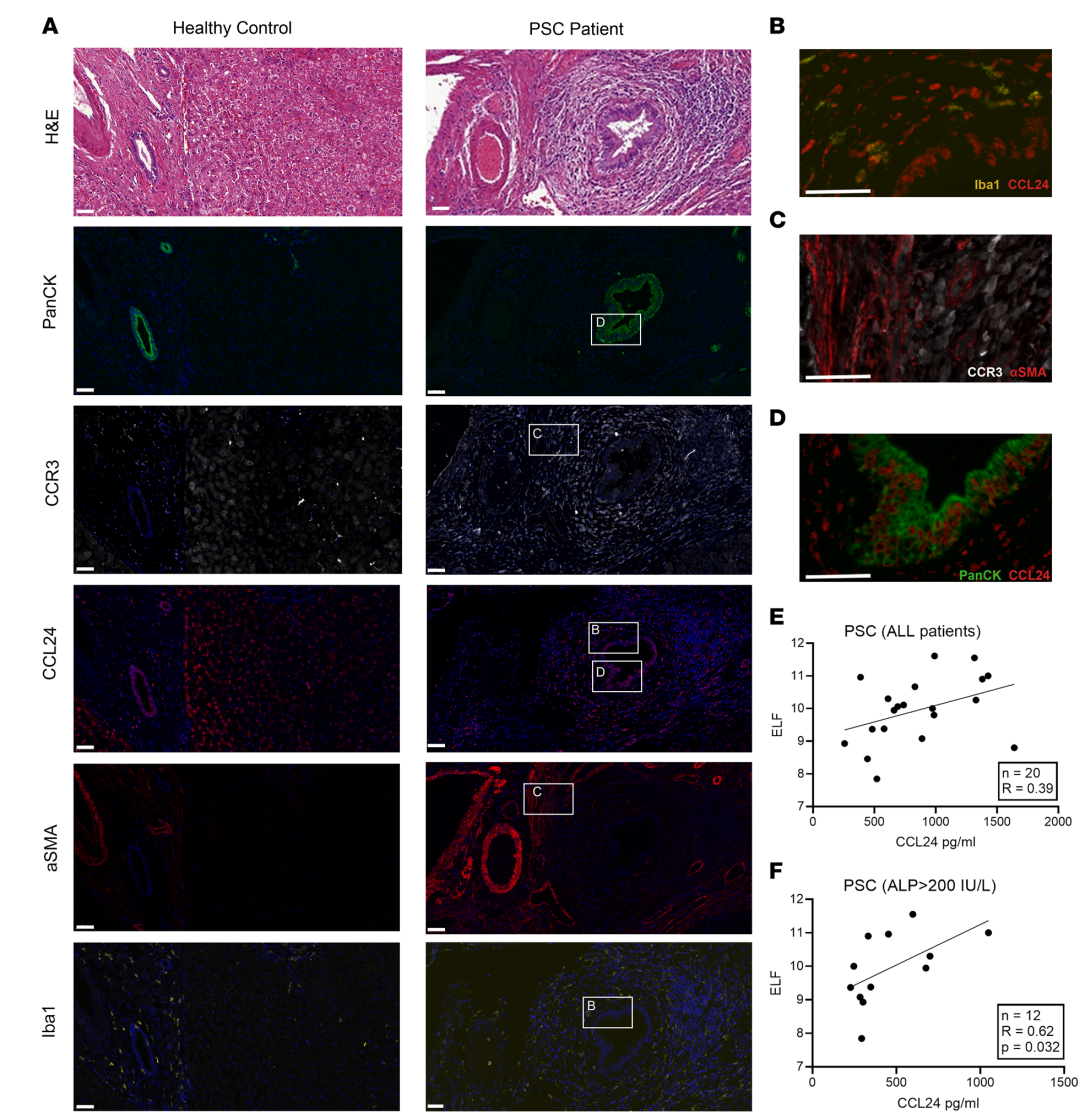
CCL24 and CCR3 are highly expressed in the bile duct areas and costained with inflammatory and fibrotic cellular markers in PSC patients’ liver biopsies. Sequential slides from bile ducts of patients with PSC and healthy controls were stained. (**A**) Representative images from healthy control and PSC patient livers stained for H&E, pan-CK, CCR3, CCL24, α-SMA, and Iba1. Staining demonstrates bile ducts surrounded by blood vessels, inflammatory cells, and activated fibroblasts. CCL24 expression is observed in cholangiocytes and in the inflammatory cells surrounding bile duct area. (**B**–**D**) High-magnification images of the biliary and peribiliary area show the colocalization of Iba1 and CCL24 (**B**), of α-SMA and CCR3 (**C**), and of pan-CK and CCL24 (**D**). Scale bar represents 50 μm. (**E**) ELF score and CCL24 levels in the serum of patients with PSC (*n* = 20) were measured for each patient. Pearson’s correlation was used to identify association of CCL24 to fibrotic biomarkers and liver damage. Dividing this cohort by ALP levels associated with higher risk of progression (**F**), resulting in higher correlation of CCL24 and the fibrotic biomarker, ELF.

To further characterize CCL24 and CCR3 spatial expression pattern in PSC human livers, and to elucidate the involvement of macrophages and cholangiocytes in CCL24 and CCR3 activity, we used immunofluorescence staining. Sequential slides of liver biopsies from healthy controls and patients with PSC were stained with H&E for general tissue and disease assessment, as well as for 2 antibody panels. The first panel was designed to demonstrate CCR3 expression and stained for CCR3, pan-CK, and α-SMA. The second panel was designed to characterize CCL24 expression and included staining for CCL24, pan-CK, and Iba1. Healthy liver sections stained for Iba1, pan-CK, and CCL24 (Figure 8A) demonstrated that, as seen in the mouse model, macrophages are present both in peribiliary and parenchymal areas. CCL24 expression in healthy livers was lower than in livers from patients with PSC (Supplemental Figure 4A). In liver samples from patients with PSC (Figure 8A), a high biliary mass was detected, as reflected by the pan-CK massive staining accompanied by immune cell infiltration and fibrosis around the bile ducts. CCL24 was expressed in cholangiocytes (Figure 8D) and in inflammatory cells surrounding the bile ducts, including peribiliary macrophages (Iba1 positive, Figure 8B and Supplemental Figure 4B), which were significantly increased in PSC. CCR3 was mainly expressed in cells surrounding the bile duct injured area, as well as in α-SMA–positive cells, reflecting its expression in activated myofibroblasts (Figure 8C and Supplemental Figure 4C).

### CCL24 correlates with fibrotic biomarkers in human patients with PSC.

The increased expression of both CCL24 and CCR3 in the fibrotic liver of patients with PSC, expressed by cholangiocytes and surrounding immune cells and in hepatic fibroblasts within the periductal fibrotic region, suggests that this CCL24/CCR3 axis might be involved in fibrosis progression/severity in patients with PSC. To better understand the relationship between CCL24 expression and progressive fibrosis in PSC, we correlated serum CCL24 levels with the validated Enhanced Liver Fibrosis (ELF) score (41). In a cohort of 20 patients with PSC (clinical characteristics are described in Supplemental Table 2), in various stages of the disease, we found that CCL24 levels positively correlated with ELF score (Figure 8, E and F). To mimic the inclusion criteria in clinical trials, we stratified patients by their ALP levels and analyzed patients with levels that exceeded 1.5 times the upper limit of normal (Figure 8F). This stratification resulted in an even stronger correlation between ELF score and CCL24. Moreover, patients presenting a combination of high ALP levels (>200 IU/L) and high CCL24 levels had an average 2-point elevation in their ELF score in comparison with individuals with low CCL24 levels (upper 25th percentile average ELF score 11.15, lower 25th percentile average ELF score 9.28, *P* ≤ 0.0229). Together, these results suggest possible upregulation of CCL24 in the fibrotic environment, as indicated by the strengthened correlation between CCL24 and fibrotic parameters as the disease progresses.

## Discussion

The pathogenesis of cholestatic liver disease involves several cell compartments that contribute to the development of chronic and progressive liver injury. In PSC, infiltrating immune cells and activation of liver myofibroblasts, along with reactive cholangiocyte proliferation, result in the destruction of bile ducts accompanied by impaired liver function (42).

Chemokines are important regulators of the immune response and, specifically in PSC, mediate cell recruitment to the liver and contribute to disease susceptibility (43–45). In this work, we focused on elucidating the role of the chemokine CCL24 (eotaxin-2) in PSC.

We show that CCL24 is overexpressed in livers of patients with PSC and specifically localized in periductal areas that drive PSC pathophysiology. Our data support CCL24 as regulator/mediator of the cholestasis/inflammatory/fibrotic axis. Expression and secretion of CCL24 in the injured sites surrounding the bile ducts by immune cells and cholangiocytes results in recruitment of specific immune cells and fibroblasts, as well as cholangiocyte proliferation and activation. The cholestasis/inflammatory/fibrotic reaction is perpetuated by a positive feedback loop that further sustains the elevation of CCL24 in the injury sites. Our data show that CCL24 regulates immune cell recruitment, specifically monocytes and macrophages, thereby enhancing the inflammatory response and the consequent tissue damage. Activated cholangiocytes interact with macrophages, activate them (including upregulation of CCR3 in macrophages), and can induce polarization toward a pro-fibrotic M2-like macrophages. Furthermore, CCL24 can directly activate fibroblasts and induce hepatic fibroblast proliferation. Neutralization of CCL24 affected 3 cellular pathways that are known to induce biliary damage: immune cell recruitment and proliferation in the liver, cholangiocyte proliferation, and fibroblast activation. This multicellular effect led to compelling improvement of PSC-related phenotypes seen in the livers of Mdr2^–/–^ mice. Nevertheless, the mouse model might not capture the whole scope of CCL24 in PSC disease. Whereas we describe the expression of CCL24 and CCR3 in human HSCs, cholangiocytes, and macrophages, in mouse liver these genes are mainly expressed only in myeloid cells (46).

Chronic inflammation is crucial for fibrosis progression and persistence, as it produces sustained signals that keep fibroblasts in an active and proliferative state and prevent their clearance from the tissue after the injury subsides. Immune cells, specifically alternatively activated (M2) macrophages, support a pro-fibrotic environment, rich in factors that activate fibroblasts and induce tolerance to prolonged activation of myofibroblasts. In this context, mechanisms promoting a sustained type 2 immune response are crucial for the perpetuation of fibrosis, shaping the inflammatory/fibrotic crosstalk (47–49). Indeed, Th2 cytokines, including IL-4 and IL-13, predominate in the biliary area of PSC and other cholestatic diseases in a manner that increases biliary inflammation (50). CCL24, secreted from liver macrophages and cholangiocytes, plays a key role in cell recruitment, contributing to a “Th2/M2-driven” vicious cycle by promoting specific migration of supportive cells to the site of inflammation. Recruited immune cells, which secrete IL-4 and IL-13, prolong the M2 polarized state of macrophages that, in turn, continue to secrete pro-fibrotic factors, specifically CCL24, creating a positive feedback loop that supports the chronic self-maintaining fibrotic environment (51). This IL-4/CCL24 relationship was demonstrated in a recent publication by Lee et al., using a model of cutaneous leishmaniasis, indicating that an IL-4/CCL24 positive feedback loop is crucial for maintaining a dominant M2 phenotype of macrophages, even in the presence of strong Th1/M1 inflammatory environments (52). Using scRNA-Seq along with spatial gene expression data from the bile duct injured areas, we revealed important information by combining expression data from specific cell populations along with their specific localization. scRNA-Seq showed that mouse liver contained several populations of macrophages. The liver-resident macrophages play a crucial role, as they act as the tissue “sensor” and are responsible for initiating a damage-related response. Measuring spatial gene expression (NanoString), we found preliminary evidence of recruitment of the resident macrophages to the injured areas surrounding the bile ducts. Genes that are unique to the parenchymal resident macrophages (e.g., Clec4f and Cd5l) were expressed in the injured peribiliary area. These resident macrophages initiate a signaling cascade that can further mediate the recruitment of monocyte-derived macrophages and drive the inflammatory response in the tissue. Inhibiting CCL24, which is a key mediator of the M2 niche, led to inhibition of the monocyte/macrophage recruitment and, subsequently, reduced accumulation of recruited and resident macrophages, which can lead to attenuation of the chronic inflammatory/fibrotic cycle (53). We also identified an interesting potentially new subpopulation of macrophage that express CCL24 as well as endothelial like genes. This population should be further examined with additional methods, to exclude technical doublets (46). Nonetheless, Burger et al. recently published single-cell data from atherosclerotic lesions that identified a similar subpopulation of “resident like macrophages,” which express CCL24 and promote atherosclerosis damage (54). Additionally, we found a change in the metabolism pattern of pan-CK–negative immune cells. This shift might be indicative of changes in immune cell polarization and activation, as demonstrated in vitro when coculturing macrophages with cholangiocytes.

We found, for the first time to our knowledge, that the role of CCL24 lies within the crosstalk between immune cells, fibroblasts, and cholangiocytes, which, together, regulate the biliary damage seen in PSC. Interestingly, Mdr2^–/–^ mouse livers showed high correlation between cholestasis, inflammation, and fibrosis markers, supporting the translational aspects of this model for drug development. Blocking CCL24 resulted in reduction of macrophage presence, cholangiocyte proliferation, and fibroblast activation that led to significant amelioration of the biliary damage. Taken together, the key role that CCL24 plays in PSC pathophysiology suggests that its blockade, using a monoclonal antibody, has the potential to serve as an effective treatment in PSC.

## Methods

### Animals.

C57BL/6 male mice, 12 weeks old, were purchased from Envigo and were acclimatized for 7 days at the animal house.

Mdr2^–/–^ mice (males and females, C57BL/6J background) were bred and tested in Hadassah Medical Center. CM-101 (D8) was administered twice weekly with subcutaneous 5 mg/kg or 10 mg/kg CM-101 (D8) from week 6, for 6 weeks. The experiment was terminated at week 13, 72 hours after the last injection. The mice were monitored for weight and general animal well-being. For serum analysis, male percentages in the test groups were 53%, 44%, and 70% for the control, 5 mg/kg, and 10 mg/kg, respectively. All other assays were performed on males only.

Sprague-Dawley male rats, 7–8 weeks old, were purchased from Sino-British SIPPR/BK and were acclimatized for 7 days at the animal house. The bile duct ligation operation was performed under ketamine/xylazine anesthesia. A midline ventral incision was made through the linea alba. Ventral midline skin and abdominal muscle wall were incised near the xiphoid process. The liver and the common bile duct were exposed. Bile duct was ligated by double ligatures, placed below the junction of the hepatic ducts and above the entrance to the pancreatic ducts. The ligatures were tightened. Then peritoneum, linea alba, and skin were closed. Animals were administrated with PBS or with CM101 10 mg/kg i.v. twice weekly. Rats were sacrificed 2 weeks after the operation.

### scRNA-Seq of Mdr2^–/–^ livers.

Livers from the mice were dissected directly on ice and chopped to 8 mm pieces, then centrifuged (60*g*, 4°C, 5 minutes). Supernatant was removed and the tissue was incubated with dissociation buffer (HBSS, 25 mg collagenase). The tissue was incubated with gentle rocking, 37°C. After 20 minutes, the cells were separated by gentle “up and down” pipetting. Cells were inspected by microscope (ZEISS) for aggregation, and in cases where aggregation was found an additional 5 minutes’ incubation was done. After a homogenous cell solution was achieved, a sample was taken for trypan blue staining to assess and count the number of living cells. The cell solution was filtered (70 μm) (Lifegene) and washed (HBSS + 4% BSA). In cases where the sample had many dead cells, a dead cell removal kit (130-090-101, Miltenyi Biotec) was used. Raw reads of each sample were processed using the “count” command of the 10x Genomics Cell Ranger software, v2.0.2, aligning the reads to the mouse mm10 (GRCm38) genome. The generated report was used for assessing the quality of the samples. The samples were analyzed by Seurat 3.0.2. Data sets were normalized using “LogNormalize,” a global-scaling normalization method.

### Kupffer cell isolation and culturing.

Kupffer cells were isolated from 12-week-old C57BL/6 mice, and livers were digested by perfusion with a collagenase solution for 10 minutes. Harvested livers were cut and cells were suspended in isolation medium (RPMI containing 1% Non-Essential Amino Acids v/v, 1% Glutamax v/v and 1% penicillin/streptomycin v/v, Thermo Fisher Scientific). Kupffer cells were purified by first eliminating hepatocytes by centrifuging them (50*g* for 2 minutes at 4°C) 4 times, taking only the nonparenchyma cells from the supernatant. Cells were then further purified by subjecting them to a density gradient (isotonic gradient 25%/50%) centrifugation (850*g*) and plating for 4 hours followed by washing of floating cells. Cells were then incubated for 24 hours with either 20 ng/mL mouse IL-4 to induce polarization or unstimulated medium (control).

### Immunofluorescence staining and immunohistochemistry.

Mouse liver tissues were trimmed, fixed in 4% neutral buffered formalin, embedded in paraffin, and sectioned at 4 μm thickness. For immunofluorescence, the sections were deparaffinized and epitope retrieval was performed. Then, sections were incubated for 1 hour with the rabbit polyclonal anti-cytokeratin (ab9377, Abcam), mouse monoclonal anti-PCNA (307902; lot: B340862, BioLegend), mouse monoclonal anti-CX3CR1 (ab184678, Abcam), and goat polyclonal anti–IBA-1 (NB100-1028, Novus Biologicals) at room temperature in a humidity chamber. Slides were then washed and incubated with secondary antibodies (donkey anti-rabbit Cy2, 711-225-152; donkey anti-rabbit Cy3, 711-545-152; donkey anti-goat Cy2, 705-225-147; donkey anti-goat Cy5, 705-175-147; donkey anti-mouse Cy3, 715-165-150; The Jackson Laboratory). Nuclei were labeled with DAPI. Images were acquired using TCS SP5 confocal laser-scanning microscope (Leica Microsystems). To assess liver collagen deposition, fixed liver sections were stained using Picrosirius red solution. For quantification of SR staining, slides were scanned using PANNORAMIC SCAN (3D Histech), and 3 randomly chosen fields from each slide were taken as snapshots at ×2 zoom (covering approximately 75%–90% of the slide). Fluorescence signal quantification for pan-CK, Iba1, and SR was done using ImageJ software (NIH). Image analysis of Iba1 and CX3CR1 fluorescence or Iba1, pan-CK, and PCNA fluorescence was performed on ×40 original magnification, ×1.2 zoom, images, using machine learning software (exemplary analysis in Supplemental Figure 1D).

### Analysis of serum biochemistry.

For serum biochemistry, blood samples were left at room temperature for 30 minutes and then centrifuged at 3,500*g* for 10 minutes at room temperature. The supernatant was collected and stored at −80°C until use. Serum levels of liver enzymes were measured using Cobas6000 (Roche Diagnostics International) and validated using LIRIS software.

### Fibrotic gene expression by real-time PCR.

Liver tissue was snap-frozen in liquid nitrogen and saved at –80°C until RNA purification with RNeasy Mini Kit (74104 QIAGEN). Briefly, 20–30 mg tissue was homogenized in RLT buffer supplemented with DTT. The lysate was centrifuged for 3 minutes at max speed, and the supernatant was mixed with 70% ethanol, loaded on a spin column, and centrifuged for 15 seconds at 8,500*g*. The column was washed once with 700 μL RW1 buffer and then twice with 500 μL RPE buffer. The RNA was eluted in 30 μL RNase-free water, and the concentration was determined by nanophotometer (Implen, NP80). cDNA synthesis was done with high-capacity cDNA reverse transcriptase (Applied Biosystems, 4368814). cDNA was diluted 1:5 and gene expression of Col1a1 and Timp1 was tested against GAPDH normalization by real-time PCR using TaqMan probes (Applied Biosystems, Mm00801666_g1, Mm01341361_m1, Mm99999915_g1). QuantStudio 1 system was used for plate reading and QuantStudio Design & Analysis software (Thermo Fisher Scientific) for results analysis.

### Spatial gene expression analysis by NanoString technology.

Gene expression of the peribiliary area of 18-week-old C57BL/6 Mdr2^–/–^ mice that were treated with 10 mg/kg CM-101 (D8) antibody (*n *= 4) or with PBS (*n *= 4) was examined by whole mouse transcriptome atlas (NanoString) according to manufacturer’s instructions. Pan-CK, F4/80, CD45, and nuclear stain were used as morphology markers. From each liver FFPE section, 3 ROIs were selected to represent the peribiliary inflamed areas (based on pan-CK and CD45 staining). All selected ROIs had similar expression levels of CD45 and F4/80. Each ROI was separated into 2 areas (pan-CK^+^ and pan-CK^–^). Counts were normalized by area size and by the third quadrantile. Cell deconvolution of the pan-CK^–^ populations was based on the immunological genomic project (ImmGen) database.

### Cell culture.

Primary human HSCs and primary human intrahepatic biliary epithelial cells (HiBECs, cholangiocytes) were purchased from ScienCell Research Laboratories (5300 & 4100, respectively). HSCs were cultured in stellate cell medium (ScienCell, 5301) supplemented with 2% fetal bovine serum (FBS), stellate cell growth supplements, and 1% penicillin/streptomycin (P/S). HiBECs were cultured in epithelial cell medium (ScienCell, 4101) supplemented with 2% FBS, epithelial cell growth supplement (ScienCell), and 1% P/S. Cells were grown in flasks precoated with poly-l-lysine and subcultured with trypsin/EDTA and trypsin neutralizing solution (ScienCell, 0103 & 0113, respectively) according to supplier protocol. LX2 cells (Merck, catalog SCC064) were cultured in DMEM, with 10% FCS. In cell culture experiments, supernatants were assayed for CCL24 using ELISA (R&D Systems, catalog DY343).

### Cholangiocytes and HSC chemokine and cytokine treatment.

Cholangiocytes were seeded in 6-well plates (50,000 cells/well in 1.5 mL full medium). The next day medium was changed to 1.5 mL starvation medium (FCS replaced by 0.1% BSA), with the following treatments in triplicate: control, TGF-β 10 ng/mL, IL-4 20 ng/mL, IL-13 100 ng/mL, IL-4+IL-13 (20 & 100 ng/mL, respectively), TNF-α 10 ng/mL, and LPS 1 μg/mL (all from Peprotech). CFSE-labeled HSCs and LX2 cells were seeded in 6-well plates (50,000 cells per well). In HSC experiments after 24 hours the medium was changed to starvation medium (0.1% BSA), with treatment with control, CCL24 (25 ng/mL or 50 ng/mL), and CCL24 + CM-101 (1 or 2.5 μg/mL). For conditioned medium experiments HSCs or LX2 cells were seeded in 6-well plates in full medium (50,000 cells per well); medium was changed after 24 hours. HSC medium was replaced to medium from M0 or M2 macrophages. LX2 medium was replaced to DMEM starvation medium or conditioned medium from M0 or M2 macrophages; IL-4 was supplemented at 20 ng/mL.

### Polarization of peripheral blood monocytes to general macrophages (M0) and polarized M1 and M2 macrophages.

Freshly drawn whole blood was diluted 1:2 in Ca-free PBS, gently placed on top of a Histopaque (MilliporeSigma) cell gradient (half the amount of diluted blood), and centrifuged for 30 minutes, at room temperature, at 400*g*. The mononuclear cell layer was washed and treated with pan-monocyte antibodies attached to MACS beads according to the kit protocol (Miltenyi Biotec). Isolated monocytes were analyzed by flow cytometry. Cells were seeded on 6-well plates in full RPMI medium, then incubated for 3 hours, after which the medium was replaced with full RPMI supplemented with 50 ng/mL human macrophage-colony stimulating factor (hM-CSF). Medium was changed again to full RPMI with 50 ng/mL hM-CSF on days 1 and 3. On day 6, the medium was changed to 1 mL full RPMI medium with (M0) 50 ng/mL hM-CSF or (M2) 50 ng/mL hM-CSF +20 ng/mL human IL-4, then incubated for 24 hours.

For coculture experiments of M0 with cholangiocytes, confluent cholangiocytes were treated for 3 days with vehicle, IL-4, or IL-13, then washed, and M0 cells were seeded on top of the cholangiocytes. After 24 hours’ incubation, media were collected for CCL24 detection, and cells were harvested for flow analysis of surface receptors. Macrophages were gated based on expression level of CD16, CCR5, and CD206.

For coculture of cholangiocytes with macrophages, cholangiocytes were labeled with 1 μM CFSE, seeded, and allowed to adhere overnight. The following day, unpolarized (M0) or polarized (M1 or M2) cells were seeded on top of the cholangiocytes. Then, 5 μg/mL of CM-101 or IgG1 isotype control was added. After 24 hours’ incubation, cells were harvested for flow analysis of Ki-67 and CFSE. Cholangiocytes were gated based on pan-CK expression.

### Tissue collection, immunohistochemistry, and immunofluorescence staining.

Paraffin-embedded liver sections from patients with PSC were obtained from the BioBank at the University College London (UCL) Institute for Liver and Digestive Health (ILDH), Royal Free Hospital. Control biopsies were from patients who, at a clinical review, had no known etiology of liver disease and normal liver histology. Staining for CCL24 and CCR3 was performed according to internal standard operating procedures at the UCL IILDH, Royal Free Hospital, using Chemomab’s proprietary monoclonal antibody, mouse anti–human CCL24, and commercial polyclonal anti–human CCR3 antibody (NBP2-15764, Novus Biologicals). Healthy liver controls, *n* = 10, and PSC livers, *n* = 10, were used. The microscope photography method was as follows. Pictures were taken using Akioskop 50 (ZEISS) at different original magnifications. IC 5 Axiocam camera (ZEISS) was used for the picture acquisitions for the evaluation. The software used was AxioVision (ZEISS). Spindle-shaped cells were identified as fibroblasts, whereas small and round mononuclear cells were identified as immune mononuclear cells. For immunofluorescence, paraffin-embedded liver sections from 10 patients with PSC and 5 healthy controls were obtained from the human biomaterial resource center, University of Birmingham, United Kingdom. After H&E staining and hepatic pathology assessment of the tissues, 5 patients with PSC and 3 healthy controls were chosen for immunofluorescence staining. The staining was done in 3-plex. Sequential slides from the same patients were stained for pan-CK, CCR3, and α-SMA or pan-CK, Iba1, and CCL24. Whole slide images were generated using a PANNORAMIC SCAN and integrated by AI-powered image quality control tools that automatically assessed focus, tissue and slide artifacts, and image quality at scale. Blinded digital segmentation and quantification of colocalized cells was performed using Reveal Biosciences imaging platform.

### Human PBMC isolation.

Patients with PSC were selected from inpatient clinics of the Institute of Gastroenterology and Hepatology at the Kaplan Medical Center in Israel (*n* = 10). Patients with PSC were identified by cholangiography demonstrating biliary structures or irregularity (consistent with PSC) and high ALP levels. Healthy donors were used as controls (*n* = 22). PBMCs were isolated from donor whole blood by Ficoll (1114544, Axis-Shield) density centrifugation. Diluted blood was added onto Ficoll at 1:2 ratio. The tube was centrifuged for 30 minutes at 425*g* at room temperature without brake. The interphase layer was collected and washed with PBS. Cells were suspended in PBS and stained with anti-CCR3 antibody (clone 61828, FAB155, R&D Systems).

### Measurement of CCL24 level in sera from patients with PSC.

Serum samples from patients with PSC (*n* = 35) were obtained from the BioBank at the UCL ILDH, Royal Free Hospital, and used to evaluate the levels of CCL24 expression. Demographics and clinical parameters such as age, sex, serum biochemistry, blood counts, and evidence of malignancy or disease-related clinical manifestations were also available for these patients. The ELF test results were available for 22 out of 35 individuals. CCL24 levels were measured by a commercial ELISA kit for human CCL24 (AB100509, Abcam). The procedure was done according to manufacturer’s instructions.

### Statistics.

Analysis of 2 groups (2-tailed *t* test or Mann-Whitney *U* test) and 1-way ANOVA with correction of multiple comparisons (summary of ANOVAs is displayed in Supplemental Table 3) were performed using Prism 9 (GraphPad) software. A *P* value of less than 0.05 was considered significant, with degree of significance indicated on the graphs.

Analysis of differentially expressed genes with the NanoString GeoMx platform was performed using linear mixed model without multiple-testing correction.

### Study approval.

All animal work was performed following approval of the National Board of Animal Studies in the Ministry of Health No-MD-18-15651-2 by the Hadassah Medical Center, Jerusalem, Israel. Animals had free access to drinking water, and food and bedding material were changed along with the cage at least twice a week.

The human PBMC study protocol was approved by the National Ethics Committee, Rehovot, Israel, according to ethics guidelines of the 1975 Declaration of Helsinki, and all patients gave their written informed consent to the study (Helsinki Approval number 0165-15-KMC). Paraffin-embedded liver sections and serum samples from patients with PSC were obtained from the BioBank at the UCL ILDH, Royal Free Hospital.

### Data availability.

The liver scRNA-Seq data of 3-month-old Mdr2^–/–^ mice generated for this study are publicly available via NCBI’s Gene Expression Omnibus (accession number GSE228596).

## Author contributions

RG, MSS, NB, IV, AP, JA, and RS designed the experiments. MSS, NB, RG, AK, OL, SA, TS, DO, IM, OH, and AS performed the experiments. PT, CJW, MP, DT, YM, and FS provided human serum samples, biopsy samples, and clinical data. AH performed the immunohistological work in human tissues. IV, RA, CJW, PT, MP, DT, JA, AP, RS, FS, YM, and AM critically reviewed the manuscript and consulted on study design. RG, MSS, and NB wrote the manuscript.

## Supplementary Material

Supplemental data

## Version 1 06/22/2023

Electronic publication
