# Supplementary material for: CCL24 regulates biliary inflammation and fibrosis in primary sclerosing cholangitis
Source: JCI Insight. 2023 Jun 22;8(12):e162270. doi: 10.1172/jci.insight.162270 (PMC10371243; doi:10.1172/jci.insight.162270)
Supplement: Supplemental data [file jciinsight-8-162270-s028.pdf]

CCL24 regulates biliary inflammation and fibrosis in primary sclerosing cholangitis

Raanan Greenman[1], Michal Segal-Salto[1], Neta Barashi[1], Ophir Hay[2], Avi Katav[1], Omer Levi[1], Ilan Vaknin[1], Revital Aricha[1], Sarit Aharoni[1], Tom Snir[1], Inbal Mishalian[2], Devorah Olam[2], Johnny Amer[3], Ahmad Salhab[3], Rifaat Safadi[3], Yaakov Maor[4], Palak Trivedi[5], Christopher Weston[5], Francesca Saffioti[6,7,8], Andrew Hall[6,7], Massimo Pinzani[6,7], Douglas Thorburn[6,7], Amnon Peled[2] and Adi Mor[1]

[1] Chemomab Therapeutics Ltd., Tel Aviv, Israel

[2] Goldyne Savad Institute of Gene Therapy, Hebrew University Hospital Jerusalem, Israel.

[3] Institute of Gastroenterology Department of Medicine, Hadassah Hebrew University Hospital, Jerusalem, Israel.

[4] Institute of Gastroenterology and Hepatology, Kaplan Medical Center, Rehovot, Israel.

[5] NIHR Biomedical Research Unit and Centre for Liver Research, University of Birmingham.

[6] UCL Institute for Liver and Digestive Health, University College of London.

[7] Sheila Sherlock Liver Centre, Royal Free London NHS Foundation Trust.

[8] Oxford University Hospitals NHS Foundation Trust

SUPLLEMENTAL MATERIAL

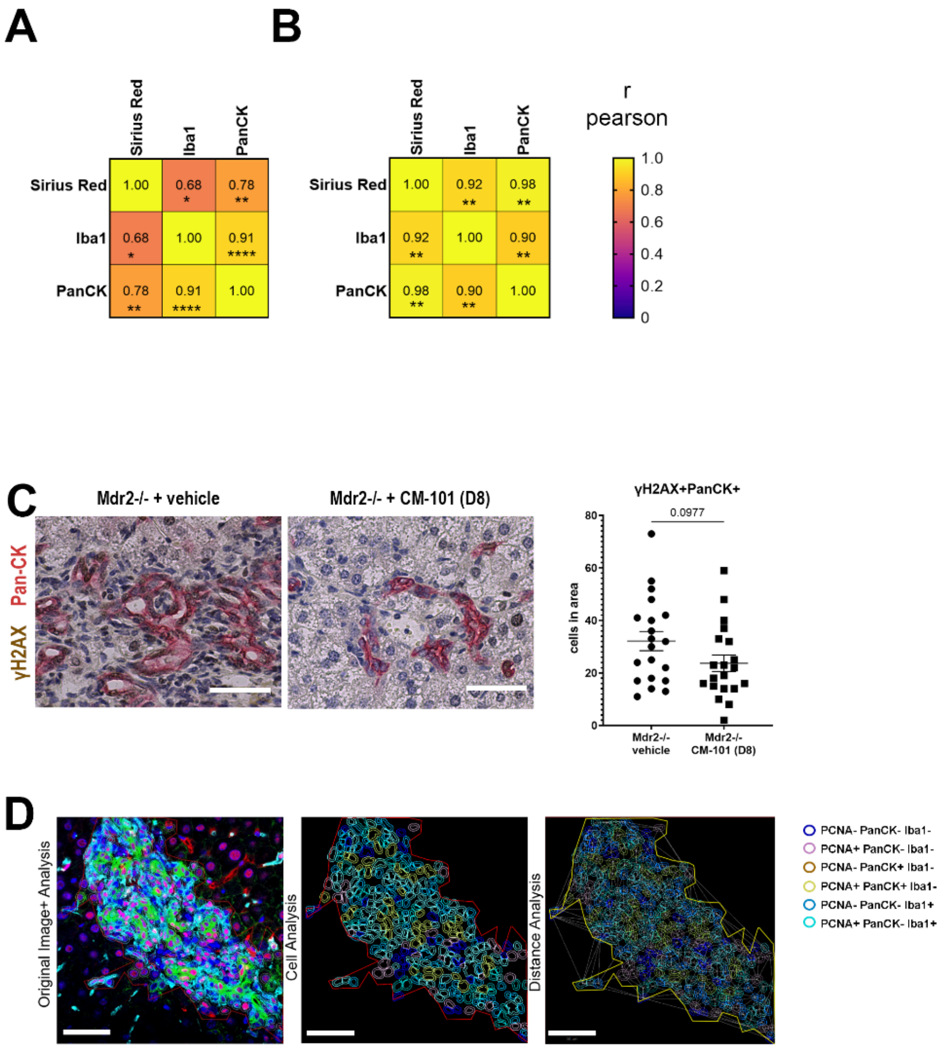

Figure S1. (**A-B**) Correlation matrices between Sirius Red positive stain area, Iba-1 positive stain area and Pan-CK positive stain area. Refers to figures 2 and 3. Analysis was performed on all animals (**A**) or on vehicle-treated animals (**B**). *$p \leq 0.05$, **$p \leq 0.01$, ***$p \leq 0.001$, ****$p \leq 0.0001$. (**C**) Biliary senescence was measured using immunohistochemistry for Pan-CK (red) and γH2AX (brown). Left graph shows quantification of Pan-CK^{+}γH2AX^{+} cells per field (N = 20 fields, 4 mice per group). Data are mean ± SEM. (**D**) Machine learning based cell detection Algorithm of IHC-F immunostaining in fibrotic area. Exemplary staining for Iba-1, Pan-CK, PCNA and DAPI. Left Panel: Original Image+ analysis (cell detection). Middle Panel: Analysis only (cell detection). Right Panel: Distance analysis (proximity between cells). Refers to Figures 2 and 4.

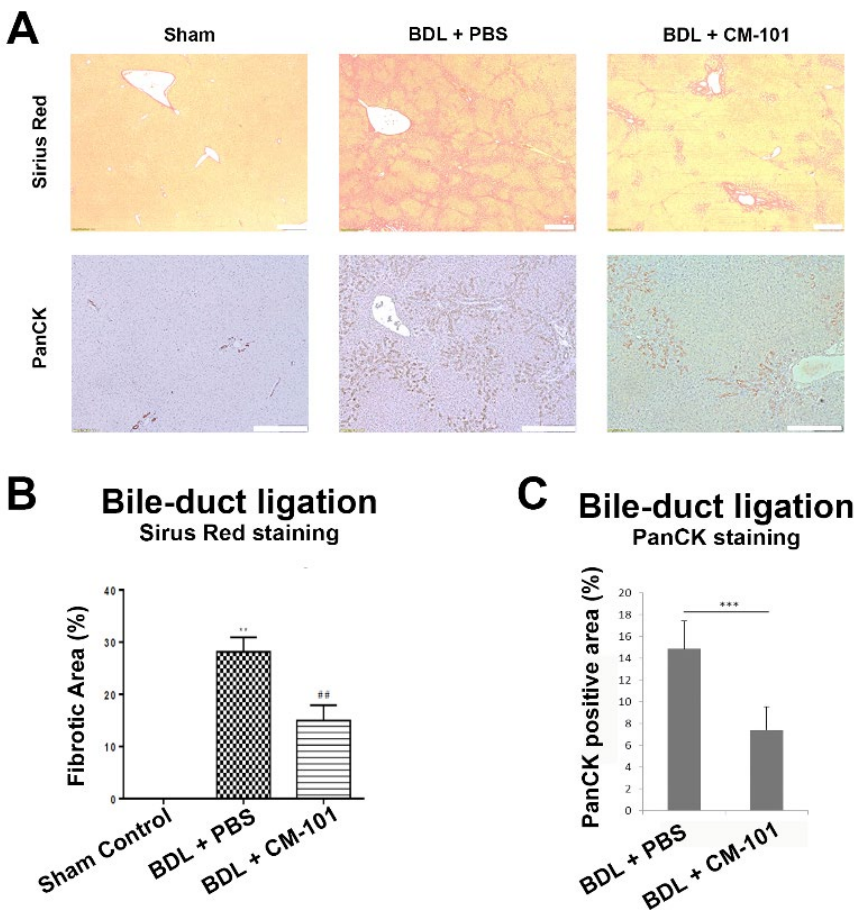

Figure S2. **CM-101 treatment improves biliary damage in bile-duct ligation (BDL) rat model.** (**A**) Representative images of Sirius Red and Pan-CK staining. Scale bar represents 200 µm. (**B**) Sirius Red quantitation. Results for sham control (N = 5), BDL with vehicle (N = 10) and BDL with CM-101 (N = 10) are expressed as mean + SEM. ** p<0.01 vs sham control; ##p<0.01 vs vehicle treated animals (BDL + PBS). Data analysis was performed by One-way ANOVA followed by Dunnett's multiple comparison test. (**C**) Quantification of Pan-CK staining in the liver of rats after ligation (N = 4). Data are mean ± SEM. Data analysis was performed by One-way ANOVA followed by Dunnett's multiple comparison test. *** p<0.001.

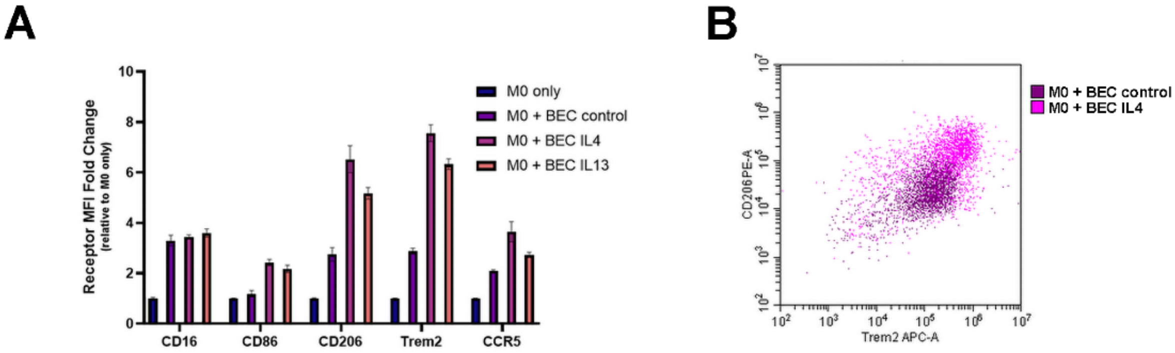

Figure S3. Cholangiocytes induce macrophage polarization towards an M2 state. Cholangiocytes were treated for 3 days with vehicle, IL-4 or IL-13, washed, and M0 cells were added on top of the cholangiocytes. (**A**) Profiling of receptor expression levels by FACS was evaluated on M0 macrophages that were grown either alone or in co-culture with pretreated BEC. Data are mean ± SEM (N = 7). (**B**) Representative dot plot of CD206 and Trem2 expression in macrophages co-cultured with cholangiocytes or with cholangiocytes that had been pretreated with IL-4. Macrophages in co-culture are gated based on expression of CCR5 and CD206.

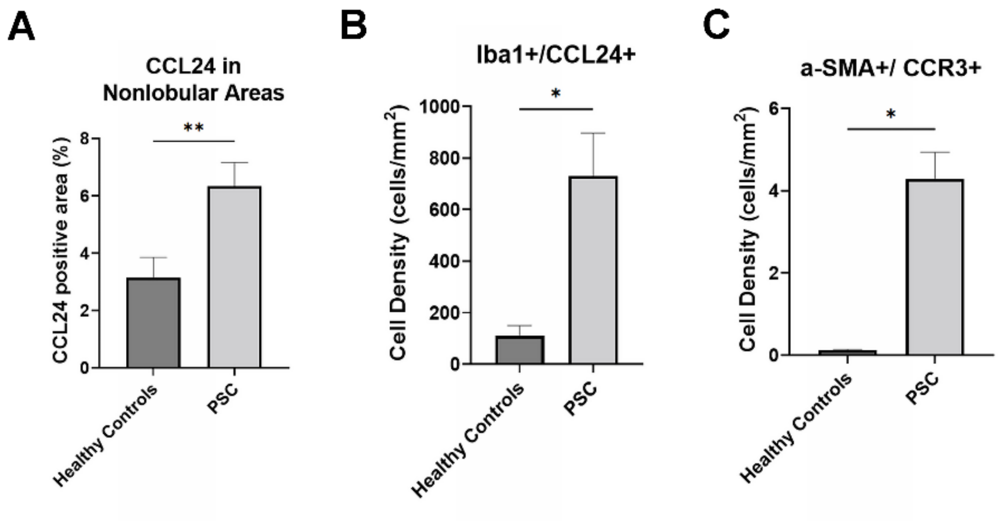

Figure S4. CCL24 and CCR3 are highly expressed in PSC patients compared to healthy controls. Refers to Figure 8. (**A**) Quantification of the area positive for CCL24 in nonlobular areas (N = 30-50 fields, 3-5 biopsies). (**B**) Quantification of cell density of Iba-1^+/CCL24^+ double-positive cells (N = 3-5). (**C**) Quantification of cell density of αSMA$^+$/CCR3^+ double-positive cells (N = 3-5). t-test *$p \leq 0.05$, **$p \leq 0.01$. All results are presented as mean ± SEM.

Table S1. Marker genes for unsupervised clustering of mononuclear phagocytes

FeatureID	Feature Name	Average	Fold Change	P-Value	Cluster	Identity
ENSMUSG00000014542	Clec4f	14.80108	4.860957	3.26E-84	1	resident macrophages
ENSMUSG00000032725	Folr2	4.191962	4.733279	5.70E-78	1	resident macrophages
ENSMUSG00000044206	Vsig4	9.871015	4.679166	6.92E-78	1	resident macrophages
ENSMUSG00000015854	Cd5l	19.92701	4.5999	2.54E-75	1	resident macrophages
ENSMUSG00000055546	Timd4	2.126358	4.56854	1.98E-70	1	resident macrophages
ENSMUSG00000026390	Marco	6.048105	4.704651	1.14E-69	1	resident macrophages
ENSMUSG00000019874	Fabp7	2.350328	4.450273	2.88E-65	1	resident macrophages
ENSMUSG00000026938	Fcna	5.462336	4.324979	5.12E-64	1	resident macrophages
ENSMUSG00000022181	C6	1.932311	3.969785	5.66E-53	1	resident macrophages
ENSMUSG00000001128	Cfp	5.252874	3.777473	1.52E-49	1	resident macrophages
ENSMUSG00000069792	Wfdc17	32.41494	3.679375	1.76E-47	1	resident macrophages
ENSMUSG00000070427	Il18bp	2.24333	3.746271	2.66E-47	1	resident macrophages
ENSMUSG00000004814	Ccl24	1.178791	4.158978	1.16E-45	1	resident macrophages
ENSMUSG00000017754	Pltp	4.201937	3.487094	1.07E-41	1	resident macrophages
ENSMUSG00000023078	Cxcl13	1.57958	4.277831	2.17E-41	1	resident macrophages
ENSMUSG00000002108	Nr1h3	1.967674	3.41858	9.60E-39	1	resident macrophages
ENSMUSG00000025993	Slc40a1	4.204657	3.360957	1.89E-38	1	resident macrophages
ENSMUSG00000025743	Sdc3	4.723325	3.20343	4.47E-35	1	resident macrophages
ENSMUSG00000004730	Adgre1	3.485594	3.215013	7.61E-35	1	resident macrophages
ENSMUSG00000034641	Cd300ld	1.35833	3.182452	9.39E-33	1	resident macrophages
ENSMUSG00000034783	Cd207	1.098996	3.567659	4.56E-32	1	resident macrophages

ENSMUSG00000059089	Fcgr4	1.215968	3.090665	4.26E-30	1	resident macrophages
ENSMUSG00000030560	Ctsc	7.856189	2.919753	6.33E-29	1	resident macrophages
ENSMUSG00000024621	Csf1r	6.246686	2.918532	9.73E-29	1	resident macrophages
ENSMUSG00000025498	Irf7	2.596061	2.885365	1.27E-27	1	resident macrophages
ENSMUSG00000036896	C1qc	17.88861	2.842053	4.15E-27	1	resident macrophages
ENSMUSG00000041827	Oasl1	2.550722	2.902105	8.76E-27	1	resident macrophages
ENSMUSG00000005413	Hmox1	3.072111	2.906486	6.40E-26	1	resident macrophages
ENSMUSG00000022180	Slc7a8	1.064539	2.814951	9.14E-25	1	resident macrophages
ENSMUSG00000030830	Itgal	1.904201	2.761804	1.74E-24	1	resident macrophages
ENSMUSG00000036887	C1qa	23.04809	2.652087	4.18E-23	1	resident macrophages
ENSMUSG00000046245	Pilra	1.17063	2.710032	9.17E-23	1	resident macrophages
ENSMUSG00000024397	Aif1	4.789519	2.4965	4.28E-20	1	resident macrophages
ENSMUSG00000036905	C1qb	22.20117	2.466634	1.21E-19	1	resident macrophages
ENSMUSG00000031613	Hpgd	3.18999	2.42775	5.38E-19	1	resident macrophages
ENSMUSG00000002602	Axl	2.628704	2.433103	7.35E-19	1	resident macrophages
ENSMUSG00000034708	Grn	5.46415	2.380216	1.64E-18	1	resident macrophages
ENSMUSG00000037697	Ddhd1	1.10353	2.464806	2.13E-18	1	resident macrophages
ENSMUSG00000043421	Hilpda	3.339606	2.411062	3.19E-18	1	resident macrophages
ENSMUSG00000019872	Smpdl3a	2.296829	2.376739	4.46E-18	1	resident macrophages
ENSMUSG00000002699	Lcp2	1.525174	2.393275	6.15E-18	1	resident macrophages
ENSMUSG00000018217	Pmp22	1.35561	2.405083	1.19E-17	1	resident macrophages
ENSMUSG00000021939	Ctsb	24.44087	2.292551	2.80E-17	1	resident macrophages
ENSMUSG00000040713	Creg1	4.319816	2.310613	3.55E-17	1	resident macrophages
ENSMUSG00000046805	Mpeg1	4.10582	2.284735	1.21E-16	1	resident macrophages
ENSMUSG00000025746	Il6	1.380092	2.510516	1.27E-16	1	resident macrophages

ENSMUSG00000026177	Slc11a1	1.304831	2.310394	2.58E-16	1	resident macrophages
ENSMUSG00000024521	Pmaip1	1.107157	2.328804	4.25E-16	1	resident macrophages
ENSMUSG00000056091	St3gal5	1.085395	2.284992	4.38E-16	1	resident macrophages
ENSMUSG00000074622	Mafb	3.422121	2.238465	2.91E-15	1	resident macrophages
ENSMUSG00000019929	Dcn	5.441714	5.398482	5.55E-35	2	Lyve1+ resident macrophages
ENSMUSG00000026043	Col3a1	2.256523	5.317688	4.05E-28	2	Lyve1+ resident macrophages
ENSMUSG00000061353	Cxcl12	1.427653	5.172832	4.05E-28	2	Lyve1+ resident macrophages
ENSMUSG00000031375	Bgn	2.869166	4.489343	9.89E-26	2	Lyve1+ resident macrophages
ENSMUSG00000070436	Serpinh1	1.072819	4.689727	9.89E-26	2	Lyve1+ resident macrophages
ENSMUSG00000018593	Sparc	4.216427	4.260605	1.18E-25	2	Lyve1+ resident macrophages
ENSMUSG00000026335	Pam	1.078363	4.697145	1.70E-25	2	Lyve1+ resident macrophages
ENSMUSG00000062960	Kdr	4.202566	4.201213	1.83E-25	2	Lyve1+ resident macrophages
ENSMUSG00000020044	Timp3	2.242662	4.313972	5.21E-25	2	Lyve1+ resident macrophages
ENSMUSG00000024065	Ehd3	4.654425	4.159644	6.19E-25	2	Lyve1+ resident macrophages

ENSMUSG00000058396	Gpr182	2.331371	4.227205	1.36E-24	2	Lyve1+ resident macrophages
ENSMUSG00000001506	Col1a1	1.136578	5.066992	1.44E-24	2	Lyve1+ resident macrophages
ENSMUSG00000056492	Adgrf5	1.998714	4.200708	1.44E-24	2	Lyve1+ resident macrophages
ENSMUSG00000020154	Ptprb	5.203309	4.062582	2.68E-24	2	Lyve1+ resident macrophages
ENSMUSG00000008575	Nfib	1.521906	4.179163	9.55E-24	2	Lyve1+ resident macrophages
ENSMUSG00000030551	Nr2f2	1.197565	4.258414	1.92E-23	2	Lyve1+ resident macrophages
ENSMUSG00000004655	Aqp1	5.926838	3.987287	4.13E-23	2	Lyve1+ resident macrophages
ENSMUSG00000026678	Rgs5	1.10054	4.671473	4.29E-23	2	Lyve1+ resident macrophages
ENSMUSG00000028763	Hspg2	1.169844	4.169912	1.09E-22	2	Lyve1+ resident macrophages
ENSMUSG00000036256	Igfbp7	7.468149	3.907942	1.34E-22	2	Lyve1+ resident macrophages
ENSMUSG00000031502	Col4a1	1.435969	4.088547	1.34E-22	2	Lyve1+ resident macrophages
ENSMUSG00000058254	Tspan7	2.328599	3.976577	2.77E-22	2	Lyve1+ resident macrophages

ENSMUSG00000029648	Flt1	1.405476	4.06456	2.77E-22	2	Lyve1+ resident macrophages
ENSMUSG00000009654	Oit3	1.452602	4.071022	3.32E-22	2	Lyve1+ resident macrophages
ENSMUSG00000027210	Meis2	1.050642	4.146498	4.27E-22	2	Lyve1+ resident macrophages
ENSMUSG00000045954	Cavin2	1.313995	4.068186	6.11E-22	2	Lyve1+ resident macrophages
ENSMUSG00000031196	F8	1.355577	4.076328	7.57E-22	2	Lyve1+ resident macrophages
ENSMUSG00000074491	Clec4g	4.817982	3.860877	1.09E-21	2	Lyve1+ resident macrophages
ENSMUSG00000020357	Flt4	1.131034	4.103544	1.19E-21	2	Lyve1+ resident macrophages
ENSMUSG00000027074	Slc43a3	1.219742	4.037388	1.49E-21	2	Lyve1+ resident macrophages
ENSMUSG00000028776	Tinagl1	1.480324	3.963193	1.56E-21	2	Lyve1+ resident macrophages
ENSMUSG00000039167	Adgrl4	1.275185	4.010228	1.90E-21	2	Lyve1+ resident macrophages
ENSMUSG00000045930	Clec14a	1.247464	4.069737	2.15E-21	2	Lyve1+ resident macrophages
ENSMUSG00000026921	Egfl7	2.2787	3.879863	3.53E-21	2	Lyve1+ resident macrophages

ENSMUSG00000063415	Cyp26b1	1.699323	3.999491	4.97E-21	2	Lyve1+ resident macrophages
ENSMUSG00000024140	Epas1	1.297362	3.921839	5.28E-21	2	Lyve1+ resident macrophages
ENSMUSG00000027800	Tm4sf1	1.671601	3.948631	5.32E-21	2	Lyve1+ resident macrophages
ENSMUSG00000054263	Lifr	2.176131	3.824845	6.21E-21	2	Lyve1+ resident macrophages
ENSMUSG00000022893	Adamts1	1.413792	4.073055	6.74E-21	2	Lyve1+ resident macrophages
ENSMUSG00000041378	Cldn5	1.02292	4.108024	7.79E-21	2	Lyve1+ resident macrophages
ENSMUSG00000050953	Gja1	1.025692	4.063663	9.93E-21	2	Lyve1+ resident macrophages
ENSMUSG00000032766	Gng11	1.940499	3.828706	1.35E-20	2	Lyve1+ resident macrophages
ENSMUSG00000031523	Dlc1	1.413792	3.83098	1.59E-20	2	Lyve1+ resident macrophages
ENSMUSG00000054072	Iigp1	4.163756	3.707892	6.04E-20	2	Lyve1+ resident macrophages
ENSMUSG00000042284	Itga1	1.17816	3.817538	1.75E-19	2	Lyve1+ resident macrophages
ENSMUSG00000026814	Eng	3.451316	3.639443	1.90E-19	2	Lyve1+ resident macrophages

ENSMUSG00000022579	Gpihbp1	2.467206	3.71451	3.03E-19	2	Lyve1+ resident macrophages
ENSMUSG00000027358	Bmp2	3.271127	3.628961	3.60E-19	2	Lyve1+ resident macrophages
ENSMUSG00000025279	Dnase1l3	4.86788	3.592317	7.44E-19	2	Lyve1+ resident macrophages
ENSMUSG00000021756	Il6st	2.988368	3.564082	7.73E-19	2	Lyve1+ resident macrophages
ENSMUSG00000009185	Ccl8	1.349785	5.131508	3.35E-18	3	monocyte derived macrophages
ENSMUSG00000016283	H2-M2	1.65268	3.917536	1.43E-31	3	monocyte derived macrophages
ENSMUSG00000024672	Ms4a7	4.015955	3.858461	6.25E-43	3	monocyte derived macrophages
ENSMUSG00000035352	Ccl12	1.287593	3.845169	1.65E-21	3	monocyte derived macrophages
ENSMUSG00000029373	Pf4	3.65202	3.737949	2.32E-34	3	monocyte derived macrophages
ENSMUSG00000052336	Cx3cr1	1.266863	3.60054	3.72E-33	3	monocyte derived macrophages
ENSMUSG00000069515	Lyz1	0.887956	3.059147	2.88E-09	3	monocyte derived macrophages
ENSMUSG00000023992	Trem2	1.995885	3.020582	8.73E-24	3	monocyte derived macrophages

ENSMUSG00000000982	Ccl3	10.0635	2.775273	2.78E-19	3	monocyte derived macrophages
ENSMUSG00000035373	Ccl7	6.464455	2.703376	1.42E-16	3	monocyte derived macrophages
ENSMUSG00000049130	C5ar1	1.969396	2.573783	4.78E-17	3	monocyte derived macrophages
ENSMUSG00000030144	Clec4d	1.201216	2.53169	2.38E-15	3	monocyte derived macrophages
ENSMUSG00000058755	Osm	0.673741	2.523479	1.64E-13	3	monocyte derived macrophages
ENSMUSG00000051439	Cd14	12.39222	2.514887	8.44E-17	3	monocyte derived macrophages
ENSMUSG00000069516	Lyz2	61.79987	2.489397	2.60E-16	3	monocyte derived macrophages
ENSMUSG00000018930	Ccl4	33.81718	2.476039	1.29E-15	3	monocyte derived macrophages
ENSMUSG00000040552	C3ar1	1.522539	2.46053	2.71E-15	3	monocyte derived macrophages
ENSMUSG00000002985	Apoe	216.3698	2.453494	2.01E-16	3	monocyte derived macrophages
ENSMUSG00000049723	Mmp12	3.182129	2.399339	8.27E-11	3	monocyte derived macrophages
ENSMUSG00000058427	Cxcl2	66.43198	2.394532	5.27E-15	3	monocyte derived macrophages

ENSMUSG00000060586	H2-Eb1	39.06544	2.374218	7.16E-15	3	monocyte derived macrophages
ENSMUSG00000036594	H2-Aa	37.46804	2.370743	6.22E-15	3	monocyte derived macrophages
ENSMUSG00000029379	Cxcl3	1.323296	2.353299	1.10E-08	3	monocyte derived macrophages
ENSMUSG00000027995	Tlr2	1.549028	2.334115	9.57E-14	3	monocyte derived macrophages
ENSMUSG00000023913	Pla2g7	1.261104	2.330939	1.12E-13	3	monocyte derived macrophages
ENSMUSG00000016529	Il10	1.739057	2.321133	1.66E-12	3	monocyte derived macrophages
ENSMUSG00000024610	Cd74	74.31878	2.317157	2.50E-14	3	monocyte derived macrophages
ENSMUSG00000038508	Gdf15	1.948665	2.252051	5.58E-11	3	monocyte derived macrophages
ENSMUSG00000043953	Ccrl2	4.458205	2.214304	2.08E-12	3	monocyte derived macrophages
ENSMUSG00000052688	Rab7b	1.059558	2.214212	4.25E-12	3	monocyte derived macrophages
ENSMUSG00000073421	H2-Ab1	35.70019	2.210538	7.81E-13	3	monocyte derived macrophages
ENSMUSG00000001348	Acp5	3.820167	2.209966	1.18E-12	3	monocyte derived macrophages

ENSMUSG00000059498	Fcgr3	1.641163	2.193414	2.93E-12	3	monocyte derived macrophages
ENSMUSG00000023349	Clec4n	1.957879	2.17395	7.79E-12	3	monocyte derived macrophages
ENSMUSG00000079547	H2-DMb1	2.409343	2.128209	1.19E-11	3	monocyte derived macrophages
ENSMUSG00000112023	Lilr4b	1.50296	2.127578	1.47E-11	3	monocyte derived macrophages
ENSMUSG00000026981	Il1rn	3.660082	2.123362	2.15E-11	3	monocyte derived macrophages
ENSMUSG00000036905	C1qb	20.58998	2.121785	6.67E-12	3	monocyte derived macrophages
ENSMUSG00000056529	Ptafr	1.555938	2.097433	3.33E-11	3	monocyte derived macrophages
ENSMUSG00000038642	Ctss	11.21519	2.077716	1.81E-11	3	monocyte derived macrophages
ENSMUSG00000036887	C1qa	19.25171	2.048065	5.24E-11	3	monocyte derived macrophages
ENSMUSG00000089929	Bcl2a1b	6.282488	2.001983	1.74E-10	3	monocyte derived macrophages
ENSMUSG00000029380	Cxcl1	9.328717	1.975358	9.70E-10	3	monocyte derived macrophages
ENSMUSG00000019122	Ccl9	2.43468	1.950773	3.37E-09	3	monocyte derived macrophages

ENSMUSG00000072620	Slfn2	4.018258	1.943894	7.48E-10	3	monocyte derived macrophages
ENSMUSG00000044811	Cd300c2	2.466927	1.928809	1.18E-09	3	monocyte derived macrophages
ENSMUSG00000024401	Tnf	4.420199	1.924498	3.43E-09	3	monocyte derived macrophages
ENSMUSG00000015396	Cd83	5.46133	1.916653	1.41E-09	3	monocyte derived macrophages
ENSMUSG00000027399	Il1a	5.915098	1.914371	1.51E-09	3	monocyte derived macrophages
ENSMUSG00000036896	C1qc	13.06366	1.909386	1.68E-09	3	monocyte derived macrophages
ENSMUSG00000040809	Chil3	2.028774	5.383906	1.11E-28	4	tissue monocytes
ENSMUSG00000029378	Areg	1.739482	4.098982	1.96E-19	4	tissue monocytes
ENSMUSG00000041324	Inhba	1.453923	4.019549	1.12E-24	4	tissue monocytes
ENSMUSG00000022018	Rgcc	1.231822	3.650665	3.94E-22	4	tissue monocytes
ENSMUSG00000029379	Cxcl3	2.683879	3.649627	1.40E-16	4	tissue monocytes
ENSMUSG00000033213	AA467197	3.361381	3.632307	1.69E-21	4	tissue monocytes
ENSMUSG00000058755	Osm	1.198227	3.50303	8.92E-21	4	tissue monocytes
ENSMUSG00000069515	Lyz1	1.243021	3.43422	1.35E-08	4	tissue monocytes
ENSMUSG00000030142	Clec4e	3.943697	3.375328	4.12E-20	4	tissue monocytes
ENSMUSG00000019122	Ccl9	4.949686	3.196459	1.17E-19	4	tissue monocytes
ENSMUSG00000027737	Slc7a11	1.498717	3.127887	2.00E-16	4	tissue monocytes
ENSMUSG00000017002	Slpi	1.834669	3.073825	5.29E-10	4	tissue monocytes

ENSMUSG00000049723	Mmp12	4.87503	3.028107	1.35E-12	4	tissue monocytes
ENSMUSG00000050335	Lgals3	14.46458	3.015229	1.37E-17	4	tissue monocytes
ENSMUSG00000049103	Ccr2	1.338207	2.972832	1.87E-15	4	tissue monocytes
ENSMUSG00000032487	Ptgs2	3.887705	2.940588	7.21E-15	4	tissue monocytes
ENSMUSG00000030789	Itgax	1.149701	2.930063	1.49E-15	4	tissue monocytes
ENSMUSG00000031444	F10	1.403531	2.910609	5.85E-15	4	tissue monocytes
ENSMUSG00000001020	S100a4	6.116184	2.887369	5.44E-14	4	tissue monocytes
ENSMUSG00000030144	Clec4d	1.584571	2.829408	2.14E-14	4	tissue monocytes
ENSMUSG00000040152	Thbs1	5.832491	2.808984	2.34E-13	4	tissue monocytes
ENSMUSG00000069516	Lyz2	80.29614	2.759482	2.00E-14	4	tissue monocytes
ENSMUSG00000056737	Capg	2.286337	2.639757	1.22E-12	4	tissue monocytes
ENSMUSG00000024659	Anxa1	1.653628	2.55727	8.67E-12	4	tissue monocytes
ENSMUSG00000027995	Tlr2	1.939187	2.546221	1.84E-11	4	tissue monocytes
ENSMUSG00000029322	Plac8	4.738783	2.531676	9.06E-11	4	tissue monocytes
ENSMUSG00000023349	Clec4n	2.562563	2.489782	6.89E-11	4	tissue monocytes
ENSMUSG00000038352	Arl5c	1.40913	2.440959	1.81E-10	4	tissue monocytes
ENSMUSG00000027398	Il1b	38.98717	2.407755	1.49E-10	4	tissue monocytes
ENSMUSG00000079293	Clec7a	5.186718	2.38309	3.49E-10	4	tissue monocytes
ENSMUSG00000035678	Tnfsf9	1.149701	2.362532	1.59E-09	4	tissue monocytes
ENSMUSG00000018927	Ccl6	4.6156	2.299958	3.16E-09	4	tissue monocytes
ENSMUSG00000046223	Plaur	3.260596	2.281232	2.77E-09	4	tissue monocytes
ENSMUSG00000051439	Cd14	12.34435	2.256193	4.11E-09	4	tissue monocytes
ENSMUSG00000026981	Il1rn	4.244187	2.211925	1.93E-08	4	tissue monocytes
ENSMUSG00000000290	Itgb2	1.640563	2.203333	7.40E-09	4	tissue monocytes
ENSMUSG00000060063	Alox5ap	2.03064	2.192362	2.78E-08	4	tissue monocytes

ENSMUSG00000058427	Cxcl2	66.89168	2.178394	2.31E-08	4	tissue monocytes
ENSMUSG00000030707	Coro1a	2.784665	2.17776	2.64E-08	4	tissue monocytes
ENSMUSG00000026193	Fn1	2.730539	2.15514	1.04E-07	4	tissue monocytes
ENSMUSG00000043953	Ccrl2	4.645463	2.091793	1.88E-07	4	tissue monocytes
ENSMUSG00000079419	Ms4a6c	2.092231	2.083584	1.68E-07	4	tissue monocytes
ENSMUSG00000056529	Ptafr	1.702154	2.081526	1.87E-07	4	tissue monocytes
ENSMUSG00000016283	H2-M2	0.920134	2.073291	1.90E-05	4	tissue monocytes
ENSMUSG00000035493	Tgfbi	2.265806	2.06463	4.38E-07	4	tissue monocytes
ENSMUSG00000041836	Ptpre	1.06198	2.051784	1.52E-07	4	tissue monocytes
ENSMUSG00000002204	Napsa	0.963061	2.04713	1.20E-06	4	tissue monocytes
ENSMUSG00000072620	Slfn2	4.626799	2.039828	2.28E-07	4	tissue monocytes
ENSMUSG00000024401	Tnf	5.098997	2.025864	6.48E-07	4	tissue monocytes
ENSMUSG00000022126	Acod1	1.646162	2.022958	1.44E-06	4	tissue monocytes
ENSMUSG00000076613	Ighg2b	2.330353	11.6269	0.000771	5	dendritic cells
ENSMUSG00000031494	Cd209a	1.070824	6.160389	3.30E-60	5	dendritic cells
ENSMUSG00000004296	Il12b	1.485675	5.869473	1.66E-29	5	dendritic cells
ENSMUSG00000031780	Ccl17	5.1759	5.85284	2.03E-50	5	dendritic cells
ENSMUSG00000037548	H2-DMb2	1.22059	5.41555	4.04E-63	5	dendritic cells
ENSMUSG00000037944	Ccr7	3.522487	5.243447	1.88E-57	5	dendritic cells
ENSMUSG00000031779	Ccl22	4.07662	5.196518	8.24E-49	5	dendritic cells
ENSMUSG00000002204	Napsa	2.301897	4.366014	2.55E-47	5	dendritic cells
ENSMUSG00000029413	Naaa	1.758248	4.18737	5.82E-42	5	dendritic cells
ENSMUSG00000051506	Wdfy4	1.235566	4.139961	7.44E-43	5	dendritic cells
ENSMUSG00000018819	Lsp1	4.185949	4.010017	1.78E-42	5	dendritic cells

ENSMUSG00000033083	Tbc1d4	2.099714	3.857039	2.06E-34	5	dendritic cells
ENSMUSG00000027447	Cst3	77.28804	3.554179	5.86E-33	5	dendritic cells
ENSMUSG00000040950	Mgl2	1.030387	3.406314	2.54E-20	5	dendritic cells
ENSMUSG00000001020	S100a4	6.755927	3.295393	9.35E-24	5	dendritic cells
ENSMUSG00000026073	Il1r2	1.021401	3.274859	1.59E-20	5	dendritic cells
ENSMUSG00000028341	Nr4a3	3.279867	3.220365	7.17E-24	5	dendritic cells
ENSMUSG00000026832	Cytip	3.477558	3.190101	1.63E-24	5	dendritic cells
ENSMUSG00000040710	St8sia4	1.298468	3.141246	2.21E-22	5	dendritic cells
ENSMUSG00000030214	Plbd1	2.942894	3.083812	1.08E-22	5	dendritic cells
ENSMUSG00000085156	Snhg15	1.075317	3.083138	4.87E-21	5	dendritic cells
ENSMUSG00000019876	Pkib	1.189139	3.013303	7.64E-21	5	dendritic cells
ENSMUSG00000000531	Grasp	1.377844	3.001097	1.70E-20	5	dendritic cells
ENSMUSG00000034634	Ly6d	1.494661	2.916121	1.21E-09	5	dendritic cells
ENSMUSG00000018821	Avpi1	1.13073	2.672699	9.37E-16	5	dendritic cells
ENSMUSG00000070691	Runx3	1.027392	2.653182	7.38E-15	5	dendritic cells
ENSMUSG00000026875	Traf1	2.129667	2.642199	4.83E-15	5	dendritic cells
ENSMUSG00000000594	Gm2a	4.31924	2.613324	1.26E-15	5	dendritic cells
ENSMUSG00000076609	Igkc	8.403349	2.540991	0.041208	5	dendritic cells
ENSMUSG00000073421	H2-Ab1	44.52681	2.535194	1.51E-14	5	dendritic cells
ENSMUSG00000060063	Alox5ap	2.25547	2.495003	1.85E-13	5	dendritic cells
ENSMUSG00000033213	AA467197	1.908014	2.488443	3.22E-10	5	dendritic cells
ENSMUSG00000028657	Ppt1	1.470698	2.455601	6.14E-13	5	dendritic cells
ENSMUSG00000030707	Coro1a	3.037247	2.440628	5.10E-13	5	dendritic cells
ENSMUSG00000001270	Ckb	3.128604	2.437539	4.59E-13	5	dendritic cells

ENSMUSG00000060586	H2-Eb1	42.2983	2.393606	1.23E-12	5	dendritic cells
ENSMUSG00000095079	Igha	1.308951	2.379745	0.195458	5	dendritic cells
ENSMUSG00000048163	Selplg	1.286487	2.371085	4.84E-12	5	dendritic cells
ENSMUSG00000048277	Syngr2	2.149137	2.330279	7.34E-12	5	dendritic cells
ENSMUSG00000037649	H2-DMa	3.413158	2.296817	1.90E-11	5	dendritic cells
ENSMUSG00000003382	Etv3	1.68037	2.246597	1.11E-10	5	dendritic cells
ENSMUSG00000022205	Sub1	5.054589	2.245928	3.82E-11	5	dendritic cells
ENSMUSG00000022876	Samsn1	1.289482	2.240562	3.09E-10	5	dendritic cells
ENSMUSG00000036594	H2-Aa	37.55223	2.233542	8.38E-11	5	dendritic cells
ENSMUSG00000006360	Crip1	13.01463	2.200013	2.51E-10	5	dendritic cells
ENSMUSG00000037894	H2az1	12.3332	2.151386	3.33E-10	5	dendritic cells
ENSMUSG00000026826	Nr4a2	1.476689	2.13373	4.15E-09	5	dendritic cells
ENSMUSG00000024610	Cd74	72.24693	2.125871	1.02E-09	5	dendritic cells
ENSMUSG00000067149	Jchain	0.569109	2.06072	0.116278	5	dendritic cells
ENSMUSG00000000682	Cd52	13.27073	2.049297	4.74E-09	5	dendritic cells

Table S2. Clinical characteristics of patients in serum analysis

N	20
Men, n (%)	10 (50%)
Age, yr, median (IQR)	49.5 (31.5-64)
IBD any, n (%)	7 (35%)
Hepatobiliary malignancies any, n (%)	0 (0%)
Fibrosis stage, median (range)	F3 (F1-F3)
ELF, median (IQR)	10 (9.2-10.9)
ALP, median (IQR)	265 (172-453)
Bilirubin, median (IQR)	12 (6.3-17)
AST, median (IQR)	48 (34.8-76)
ALT, median (IQR)	63.5 (46-148)
Creatinine, median (IQR)	69 (57.3-77)
PLT, median (IQR)	268 (223-360)
WCC, median (IQR)	7.2 (5.2-7.6)
INR, median (IQR)	1 (1-1.1)

Table S3. ANOVA followed by Holm-Šídák's multiple comparisons test Statistical Analysis

**ANOVA summary**	**F**	**P value**	**P value summary**
ALT	9.849	<0.0001	****
ALP	7.199	0.0003	***
BA	6.624	0.0007	***
Col1A1	4.668	0.0133	*
Timp1	4.318	0.018	*
LX2 CCL24 RNA	45.25	<0.0001	****
BEC CCL24 RNA	40.68	<0.0001	****
BEC CCL24 ELISA	111.1	<0.0001	****
HSC proliferation by CFSE	15.47	0.0005	***
HSC proliferation by cell number	25.78	<0.0001	****
LX2 proliferation	19.43	<0.0001	****
BEC proliferation by CFSE: Co-culture	1.58	0.2197	ns
BEC proliferation by CFSE: CM-101 treatment	36.11	<0.0001	****
BEC proliferation by CFSE: Interaction	4.12	0.0171	*
BEC proliferation by Ki67: Co-culture	24.68	0.0998	ns
BEC proliferation by Ki67: CM-101 treatment	96.96	<0.0001	****
BEC proliferation by Ki67: Interaction	2.33	<0.0001	****
**Holm-Šídák's multiple comparisons test**	**Mean Diff.**	**Adjusted P Value**	**P value Summary**
<u>ALT</u>			
Mdr2-/- vs. WT	160.9	<0.0001	****

Mdr2-/- vs. Mdr2-/- 5mg/kg	24.75	0.3001	ns
Mdr2-/- vs. Mdr2-/- 10mg/kg	58.35	0.0247	*
ALP			
Mdr2-/- vs. WT	38.51	0.0026	**
Mdr2-/- vs. Mdr2-/- 5mg/kg	17.65	0.0505	ns
Mdr2--/ vs. Mdr2-/- 10mg/kg	33.61	0.0006	***
BA			
Mdr2-/- vs. WT	24.96	0.0012	**
Mdr2-/- vs. Mdr2-/- 5mg/kg	13.74	0.0081	**
Mdr2-/- vs. Mdr2-/- 10mg/kg	10.2	0.0263	*
Col1A1			
Mdr2-/- vs. Mdr2-/- 5mg/kg	0.1444	0.2756	ns
Mdr2-/- vs. Mdr2-/- 10mg/kg	0.4091	0.0069	**
Timp1			
Mdr2-/- vs. Mdr2-/- 5mg/kg	-0.02826	0.8737	ns
Mdr2-/- vs. Mdr2-/- 10mg/kg	0.4851	0.019	*
BEC CCL24 RNA			
control vs. IL-4	-13.97	<0.0001	****
control vs. IL-13	-11.78	<0.0001	****
BEC CCL24 ELISA			
control vs. IL-4	-11.64	<0.0001	****

control vs. IL-13	-11.27	<0.0001	****
control vs. TGF-b	1.13	0.37	ns
control vs. LPS	-0.08633	0.9203	ns
control vs. TNF-a	1.67	0.2	ns
<u>LX2 proliferation</u>			
Control vs. CCL24 25ng/ml	-0.1574	0.0047	**
Control vs. CCL24 25ng/ml+ CM-101 1ug/ml	-0.03845	0.8394	ns
Control vs. CCL24 25ng/ml+ CM-101 2.5ug/ml	-0.0021	0.95	ns
Control vs. CCL24 50ng/m	-0.2548	<0.0001	****
Control vs. CCL24 50ng/ml+ CM-101 1ug/ml	-0.1344	0.0146	*
Control vs. CCL24 50ng/ml+ CM-101 2.5ug/ml	0.01791	0.9357	ns
CCL24 25ng/ml vs. CCL24 25ng/ml+ CM-101 1ug/ml	0.1189	0.0282	*
CCL24 25ng/ml vs. CCL24 25ng/ml+ CM-101 2.5ug/ml	0.1553	0.005	**
CCL24 25ng/ml vs. CCL24 50ng/m	-0.09744	0.0897	ns
CCL24 25ng/ml vs. CCL24 50ng/ml+ CM-101 1ug/ml	0.02301	0.9357	ns
CCL24 25ng/ml vs. CCL24 50ng/ml+ CM-101 2.5ug/ml	0.1753	0.0018	**
CCL24 25ng/ml+ CM-101 1ug/ml vs. CCL24 25ng/ml+ CM-101 2.5ug/ml	0.03635	0.8394	ns
CCL24 25ng/ml+ CM-101 1ug/ml vs. CCL24 50ng/m	-0.2164	0.0002	***
CCL24 25ng/ml+ CM-101 1ug/ml vs. CCL24 50ng/ml+ CM-101 1ug/ml	-0.09591	0.0897	ns
CCL24 25ng/ml+ CM-101 1ug/ml vs. CCL24 50ng/ml+ CM-101 2.5ug/ml	0.05636	0.555	ns
CCL24 25ng/ml+ CM-101 2.5ug/ml vs. CCL24 50ng/m	-0.2527	<0.0001	****
CCL24 25ng/ml+ CM-101 2.5ug/ml vs. CCL24 50ng/ml+ CM-101 1ug/ml	-0.1323	0.0153	*
CCL24 25ng/ml+ CM-101 2.5ug/ml vs. CCL24 50ng/ml+ CM-101 2.5ug/ml	0.02001	0.9357	ns
CCL24 50ng/m vs. CCL24 50ng/ml+ CM-101 1ug/ml	0.1205	0.0282	*
CCL24 50ng/m vs. CCL24 50ng/ml+ CM-101 2.5ug/ml	0.2727	<0.0001	****

CCL24 50ng/ml+ CM-101 1ug/ml vs. CCL24 50ng/ml+ CM-101 2.5ug/ml	0.1523	0.0055	**
HSC proliferation by CFSE			
control vs. CCL24	-0.1594	0.0007	***
control vs. CCL24+CM-101	-0.01032	0.7888	ns
CCL24 vs. CCL24+CM-101	0.149	0.0038	**
HSC proliferation by cell number			
control vs. CCL24	-0.5172	<0.0001	****
CCL24 vs. CCL24+CM-101	0.4792	0.0003	***
LX2 CCL24 RNA			
control vs. IL4	-5.477	<0.0001	****
control vs. M0-CM	-0.02823	0.9680	ns
control vs. M0-CM + IL4	-3.858	0.0013	**
control vs. M2-CM	-7.892	<0.0001	****
IL4 vs. M0-CM	5.448	<0.0001	****
IL4 vs. M0-CM + IL4	1.619	0.1220	ns
IL4 vs. M2-CM	-2.415	0.0285	*
M0-CM vs. M0-CM + IL4	-3.830	0.0006	***
M0-CM vs. M2-CM	-7.864	<0.0001	****
M0-CM + IL4 vs. M2-CM	-4.034	0.0011	**
